# Functionalized Nanomaterials Capable of Crossing the Blood–Brain Barrier

**DOI:** 10.1021/acsnano.3c10674

**Published:** 2024-01-09

**Authors:** Shuai Zha, Haitao Liu, Hengde Li, Haolan Li, Ka-Leung Wong, Angelo Homayoun All

**Affiliations:** † Hubei University of Chinese Medicine, School of Laboratory Medicine, 16 Huangjia Lake West Road, Wuhan 430065, China; ‡ Hong Kong Baptist University, Department of Chemistry, Ho Sin Hang Campus, 224 Waterloo Road, Kowloon, Hong Kong SAR 999077, China; § Dalian University of Technology School of Chemical Engineering, Lingshui Street, Ganjingzi District, Dalian 116024, China; ∥ The Hong Kong Polytechnic University Department of Applied Biology and Chemical Technology, Building Y815, 11 Yuk Choi Road, Hung Hom, Kowloon, Hong Kong SAR 999077, China; △ Hubei Shizhen Laboratory, Wuhan 430061, China

**Keywords:** functionalized nanomaterials, BBB crossing, receptor-mediated transcytosis, absorptive-mediated transcytosis, BBB receptor-mediated endocytosis, noninvasive BBB cargo delivery, central nervous system delivery, brain delivery, neurotheranostics

## Abstract

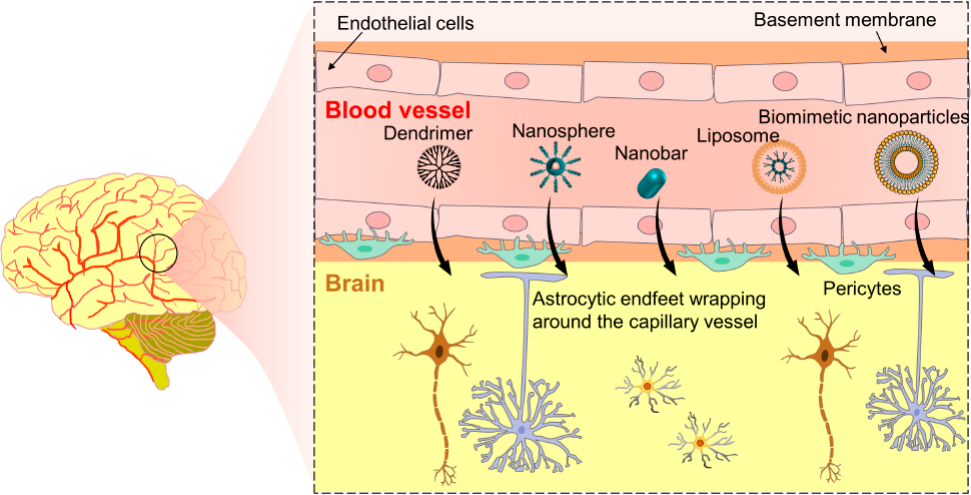

The blood–brain barrier (BBB) is a specialized semipermeable structure that highly regulates exchanges between the central nervous system parenchyma and blood vessels. Thus, the BBB also prevents the passage of various forms of therapeutic agents, nanocarriers, and their cargos. Recently, many multidisciplinary studies focus on developing cargo-loaded nanoparticles (NPs) to overcome these challenges, which are emerging as safe and effective vehicles in neurotheranostics. In this Review, first we introduce the anatomical structure and physiological functions of the BBB. Second, we present the endogenous and exogenous transport mechanisms by which NPs cross the BBB. We report various forms of nanomaterials, carriers, and their cargos, with their detailed BBB uptake and permeability characteristics. Third, we describe the effect of regulating the size, shape, charge, and surface ligands of NPs that affect their BBB permeability, which can be exploited to enhance and promote neurotheranostics. We classify typical functionalized nanomaterials developed for BBB crossing. Fourth, we provide a comprehensive review of the recent progress in developing functional polymeric nanomaterials for applications in multimodal bioimaging, therapeutics, and drug delivery. Finally, we conclude by discussing existing challenges, directions, and future perspectives in employing functionalized nanomaterials for BBB crossing.

## 1 Introduction

The interchange of biological materials that are indispensable for survival and function of neurons in the central nervous system (CNS) is well-controlled by the blood–brain barrier (BBB). The BBB is the largest interface for blood and brain exchanges. It establishes and maintains a very intricate equilibrium and homeostasis within the CNS parenchyma. Understandably, the structural integrity of the BBB is crucial for coherent function of the brain. Evidently, such superspecialized permeability constrains the therapeutic efficacy of many potential pharmacological agents that have been developed and could be used to treat neurological disorders as well. Recently, various methods of minimally invasive delivery have gained extensive attention and have become among the most prominent therapeutic strategies for CNS diseases. Nonetheless, any such strategy needs to overcome anatomical and physiological barriers in the CNS safely and effectively.^1^

The BBB is composed of endothelial cells, pericytes, astrocytes, and basement membrane, which form a highly selective semipermeable membrane. The main function of the BBB is to regulate the exchange of substances between the blood and the CNS based on brain physiological needs. Briefly, the BBB shields the CNS from toxins, pathogens, and foreign substances in the blood. It is also the gateway to supply the brain tissues with nutrients and expel unnecessary materials from the brain back into the bloodstream. The BBB establishes a very delicate ion homeostasis and adjusts the level of neurotransmitters, which guarantee impeccable function of the CNS.^2^ Understandably, this peculiar superselective barrier also prevents blood circulating macromolecules such as recombinant proteins, antibodies, and nucleic acids,^3^ as well as micromolecules, like trastuzumab,^4^ doxorubicin,^5^ or vincristine,^6^ from entering into the brain parenchyma. However, development of nanotechnology has made significant breakthroughs in creating reliable gates through the BBB.^7^ Since, nanomaterials can also deliver loaded agents (drugs, genes, etc.), they have been exploited as multifunctional delivery systems to the CNS due to their innocuous ability to cross the BBB.^8^

**Figure 1:**
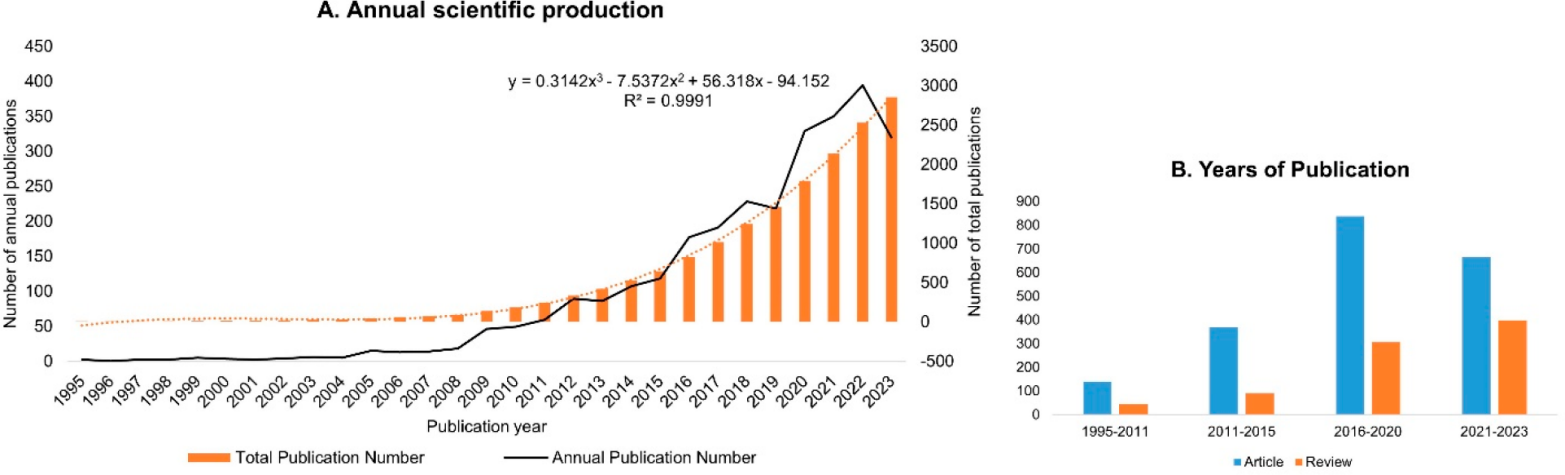
Number of publications on nanoparticles crossing the BBB: Number of publications containing the terms (“nanoparticle*” OR “nanomaterial*” OR “nanocarrier*”) AND (“BBB” OR “blood*brain*barrier”) since 1995. (A) Annual number of articles, reviews, and proceedings was analyzed. The orange box plot represents total number of publications; the orange dashed line represents the trendline, and the black line represents the annual number of publications. (B) Number of published articles and reviews on functionalized nanoparticles crossing the BBB from 1995 to 2023. Blue bars represent published articles, and orange bars represent published reviews. Source: Web of Science, as assessed in October 2023. Note that papers in the categories “instruments instrumentation”, “metallurgy metallurgical engineering”, “robotics”, “energy fuels”, and “engineering mechanical” were excluded.

Nanomaterials like inorganic nanoparticles, liposomes, dendrimers, and polymers have gained extensive attention in the field of drug and gene delivery, due to their incomparable advantages, such as higher drug loading capacity, lower dose and administration frequency, good biocompatibility (less side effects), high stability, enhanced bioavailability and biodegradability, targeted applications with less invasive theranostic techniques, decreased acquired drug resistance, negligible toxicity and immunogenicity, reduced off-target toxicity, feasibility for various routes of administration (intranasal, oral, and intravenous, and intramuscular injection), sustained and controlled drug release, unique properties (physical, electronic, chemical, and optical), and effective tracing of drug delivery.^3,9^ These nanomaterials are being designed and developed as safe, effective, feasible, and practical tools for diagnosing and treating diseases of the CNS.^10^ The current interest in functionalized NPs crossing the BBB is reflected in the number of publications per year, which showed a relatively stable growth trend between 1995 and 2023 in general (Figure 1A). Papers published in the last 3 years account for 37% of the total number. According to the quantitative analysis of the chart, a growth trend was revealed in the past few decades in global original research, from 140 articles between 1995 and 2011 to 837 articles from 2016 to 2020 and 666 articles in the past three years (2021–2023). In addition, the research results indicate that functionalized NPs crossing the BBB have attained increasing attention and discussion, from 46 reviews during 1997–2011 to 306 reviews in 2016–2020 and 398 reviews in just 2021–2023 (Figure 1B). This rapid growth in published reviews highlights the emerging promise yet continued challenges of brain drug delivery using NPs. In this context, this review article first introduces the anatomical structure and physiological function of the BBB and then expounds on the challenges of BBB crossing, describing recent discoveries and innovative solutions. The mechanisms for BBB crossing of various nanomaterials and their cargos are also explained. Moreover, we highlight the advantages of implementing nanomaterials as carriers for drug delivery to the CNS via BBB crossing and the latest progress in these fields.

## 2 Anatomical Structure of Intact BBB

**Figure 2:**
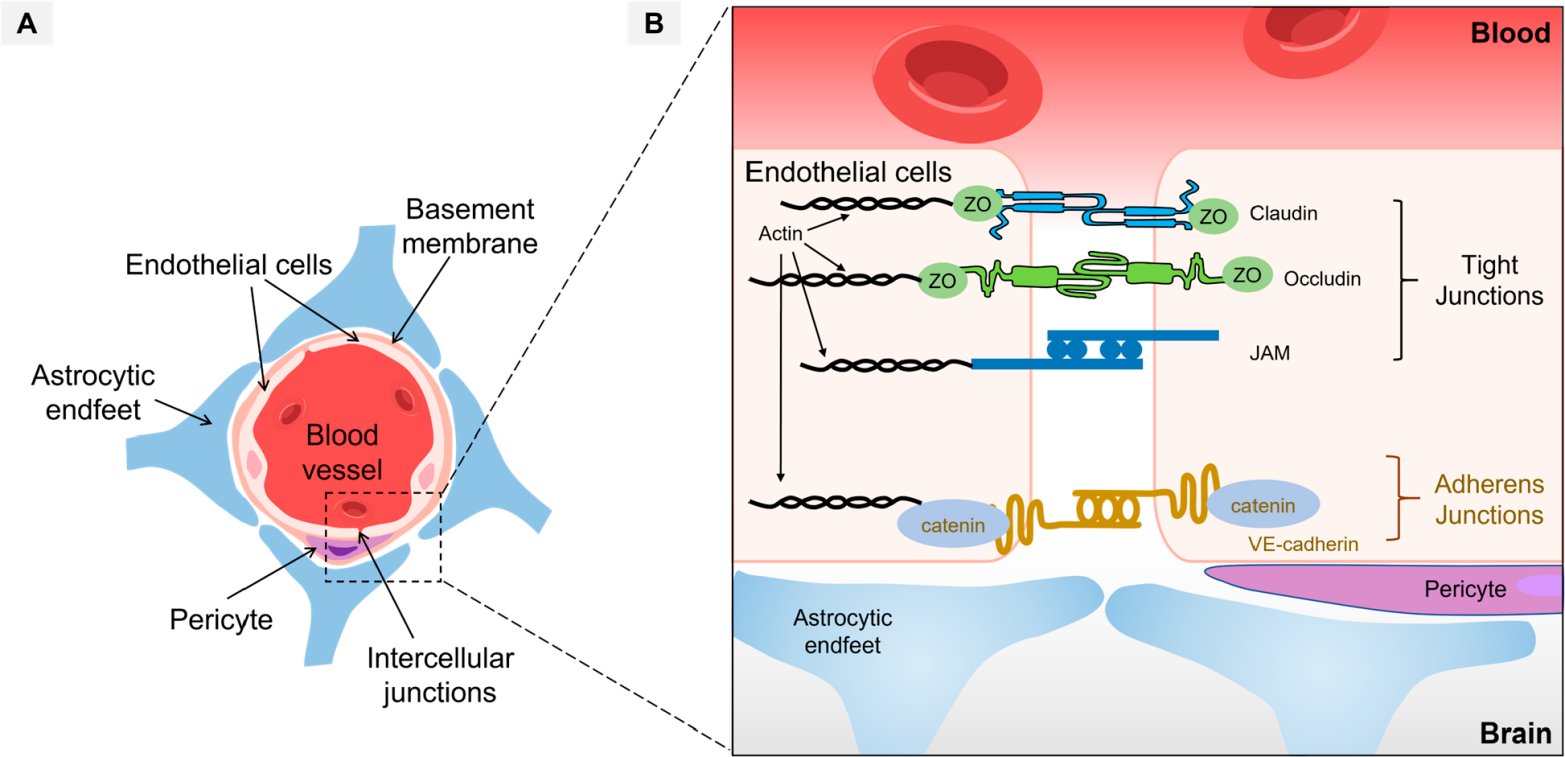
Blood–brain barrier (BBB) and intercellular junctions between endothelial cells. (A) Structures of the BBB, which is composed of the endothelial cells, pericytes, basement membrane, and astrocytes; (B) Schematic of brain endothelial connections, including tight junctions, gap junctions, and adherens junctions. Reproduced with permission under a Creative Commons License from ref (21). Copyright 2021 the Authors, published by Springer Nature.

The BBB is composed of microvascular endothelial cells, pericytes, and basement membrane from one side and long foot shaped extensions of astrocytes from the other side, forming an intimate association of end-feet protrusions of astrocytes that wrap around the endothelial cells of the capillary microvascular (Figure 2A).^11,12^

### 2.1 Endothelial Cells

Endothelial cells (ECs) are the basic building blocks of the BBB endothelium and are characterized by a flattened appearance in the inner layer.^13^ They differ morphologically and metabolically from those in other mammalian capillary endothelia, containing a higher concentration of mitochondria and no fenestrations, forming a physically restrictive barrier by intercellular junctions.^14^ The cerebral ECs have very few pinocytotic vesicles, yet they transport nutrients from the blood to the brain mainly via energy-dependent (produced by mitochondria) active transport. The BBB permeability can be altered or disrupted by mitochondria loss or any other dysfunction in the cerebral ECs.^15,16^ In addition, the cerebral ECs offer an enzymatic barrier due to the presence of proteolytic enzymes including γ-glutamyl transpeptidase, alkaline phosphatase, and aromatic acid decarboxylase. This enzymatic barrier takes part in the assimilation of the neuroactive blood-borne solutes and drugs.^17^

### 2.2 Pericytes and Vascular Basement Membrane

Pericytes (PCs) and vascular basement membrane (BM) lie in the middle layer of the BBB. PCs are partly embedded within the BM and make peg-and-socket junctions with ECs through adhesion molecules.^18^ These peg–socket junctions are formed where the BM is missing and act as anchors in which either pericytes or endothelial cell cytoplasmic projections (peg) lock into introversions (socket) of the other component.

### 2.3 Astrocytes

Astrocytes are the most abundant glial cells in the CNS. They are star-shaped and possess fine dendrite-like structures. At the end of these dendritic processes, there are small swellings called foot processes. There are two types of astrocytes: protoplasmic (located in the gray matter) and fibrous (located in the white matter). Astrocytes are the primary supporting cells of the nervous system, providing structural and metabolic support to the neurons, and have a vital role in tissue repair processes in the CNS. Interestingly, astrocytes also participate in the formation of tight junctions between cells lining the capillaries in the brain, resulting in the formation of the BBB membrane.^19^ Clearly, any dysfunction or malfunction of astrocytes would lead to the obliteration of tight junction protein expression and leakage of substances.^20^

### 2.4 Junctions at the Blood–Brain Barrier

Brain ECs are closely connected by two distinct types of junctions: tight junctions and adherens junctions (Figure 2B).^21^ Tight junctions consist of many different proteins in the cerebral microvasculature, including specific transmembrane and intracellular proteins. The transmembrane proteins comprise junction adhesion molecules (JAMs), claudins 1, 3, 5, and 12, and occludins, and the intracellular proteins include zonula occludens-1 (ZO-1). Adherens junctions are formed by cadherins (VE-cadherin or cadherin-5) and catenins, which are present at the basal side of EC–EC contacts. The primary mechanical role of catenins is to connect cadherins to actin filaments.^22^ Furthermore, the endothelial Wnt/β-catenin pathway has been found to be one of the major molecular drivers of barriergenesis in the brain.^23,24^ Hussain et al.^25^ demonstrated the importance of β-catenin in maintaining BBB integrity using endothelial-specific β-catenin conditional knockout mice. These mice exhibited severe leakage of plasma proteins IgG and albumin into the cerebral cortex. This breakdown of the BBB was attributed to both paracellular and transcellular transport dysfunction. Mechanistically, the loss of β-catenin led to downregulation of TJ proteins, physical disruption of intercellular junctions, decreased expression of Mfsd2a (an inhibitor of transcytosis), and increased caveolin-1 (a protein associated with endocytosis).^25^

Owing to their components and biological functions, tight junctions and adherens junctions together maintain microvascular integrity and control the transport of molecules.^26^ However, the restrictive properties of the BBB prevent most therapeutic agents from entering the brain parenchyma from the blood capillary vasculature. Approximately 98% of small molecules and nearly all large pharmacological and other molecules, such as polypeptides, antibodies, or oligonucleotides, cannot cross the BBB.^27^ The protective function of the BBB is also one of the major limitations in drug delivery into the CNS and treatment.

## 3 Abnormal BBB Structure in CNS Diseases

BBB disruption typically involves degradation or shrinkage of ECs and altered paracellular transport pathways through reduced expression of TJ proteins and/or TJ translocations.^28^ In addition, the expression or function of BBB-associated receptors and transport machinery may be affected, resulting in dysregulated molecular transport.^29^ Importantly, disruption of the BBB may also involve different cellular components beyond the brain endothelium. These include pericyte degeneration or decreased pericyte coverage, basement membrane alterations,^30^ and detachment of astrocyte terminal feet from the vascular basement membrane.^31^ These astrocyte terminal podia may also exhibit a swollen phenotype under certain pathological conditions.^32^ Besides, ECs can increase the expression of leukocyte adhesion molecules, leading to increased leukocyte extravasation into the brain parenchyma under pathological conditions.^33^ These leukocyte–EC interactions can also directly contribute to the increased BBB permeability through the release of reactive oxygen species, cytokines, and other barrier-damaging mediators.^21^ Understanding BBB dysfunction in CNS diseases may reveal opportunities for improvement in diagnosis, monitoring, and disease-modifying interventions.

### 3.1 BBB in Stroke

**Figure 3:**
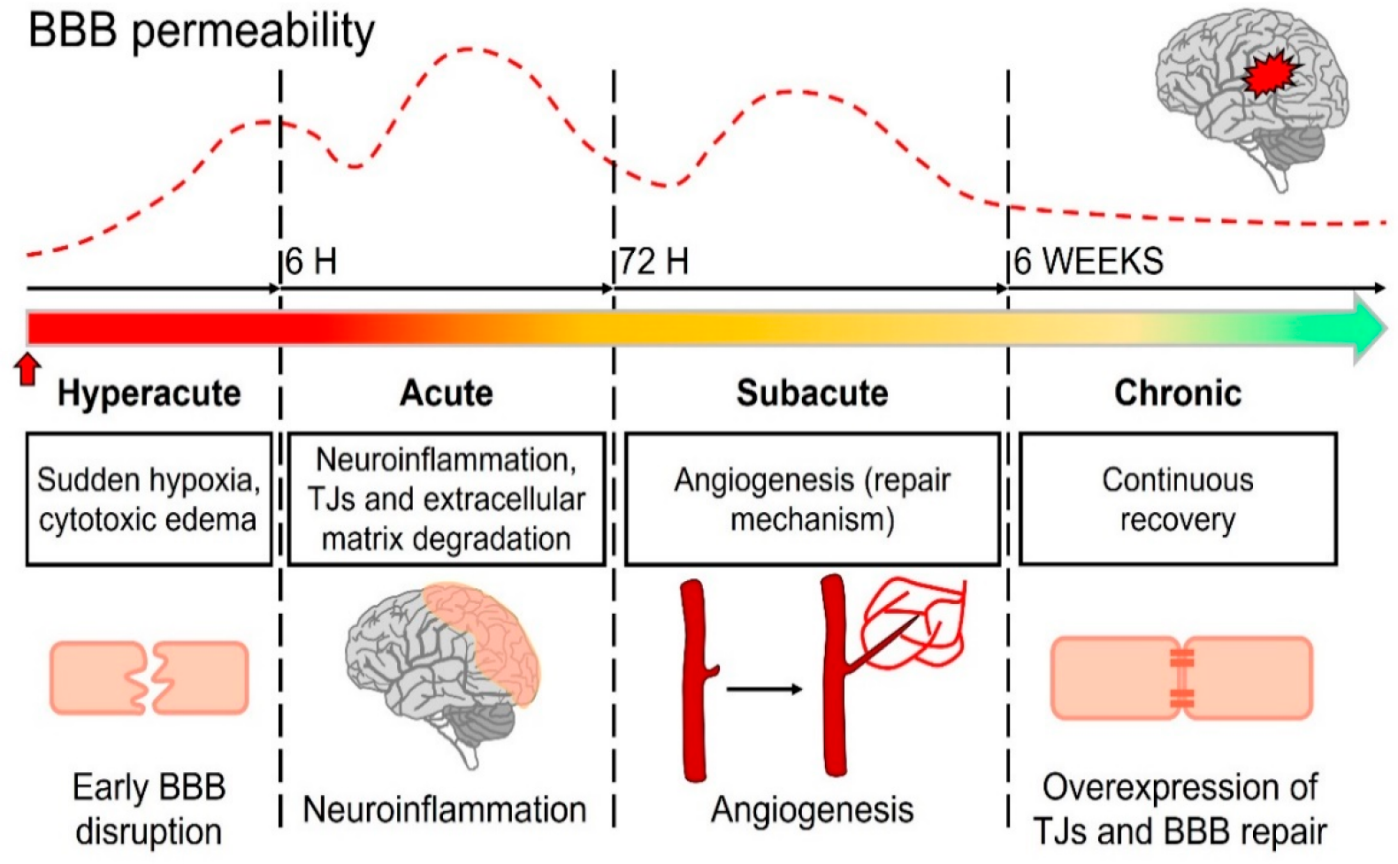
Evolution of blood–brain barrier permeability in four phases of stroke and major pathophysiological processes in each phase. Early BBB disruption occurs in the hyperacute phase. The acute phase occurs thereafter, and neuroinflammation is a major factor in the development of injury. The subacute and chronic phases are characterized by repair processes, mainly angiogenesis and restoration of BBB. Reproduced with permission under a Creative Commons License from refs (37 and 39). Copyright 2020 and 2021 the Authors, published by Frontiers Media SA and Multidisciplinary Digital Publishing Institute.

Stroke is a cerebrovascular disease that seriously threatens human health. There are two types of strokes: ischemic and hemorrhagic. Ischemic stroke accounts for the majority of cases (80–87%).^34^ BBB permeability appears to follow multiphasic patterns at different stroke stages that are associated with different biological substrates (Figure 3). In the hyperacute phase (first 6 h), sudden hypoxia disrupts the BBB, leading to cytotoxic edema, reactive oxygen species (ROS) generation, TJ and extracellular matrix degradation, and increased permeability; in the acute phase (6–72 h), the neuroinflammatory response exacerbates the BBB injury through the downregulation and degradation of TJs and extracellular matrix, gliosis, and EC activation, thereby causing increased permeability and subsequent hemorrhagic transformation risk by reperfusion therapy; in the subacute phase (72 h to 6 weeks), major repair mechanisms, such as angiogenesis, occur. Brain recovery and BBB permeability are closely associated with lesion volumes and stroke severity,^35^ as well as neuroinflammation involving activated microglia and inflammatory cytokines. Bernardo-Castro et al.^36^ (2023) found that BBB permeability was highest at 3–10 days poststroke possibly due to regenerative mechanisms, such as vascular remodeling, and this time window of increased permeability correlated with better clinical recovery.^35,36^ During the chronic phase (>6 weeks), an increase in BBB restoration factors restores the barrier permeability through the overexpression of TJ proteins, reorganization of junction proteins, and secretion of neurotrophic factors.^37^

In short, BBB disruption, one of the major pathophysiological features of stroke, is closely associated with TJ dysfunction, endothelial injury, retraction of astrocytic endfeet, and disruption of cell–cell interaction.^38^ In the early phases of stroke, NPs can easily penetrate the brain due to the high permeability of the injured BBB. In the chronic phase, vasculogenesis usually occurs along with partial restoration of BBB integrity, therefore impairing NP transport through leaky vessels.^39^

### 3.2 BBB in Brain Tumors

**Figure 4:**
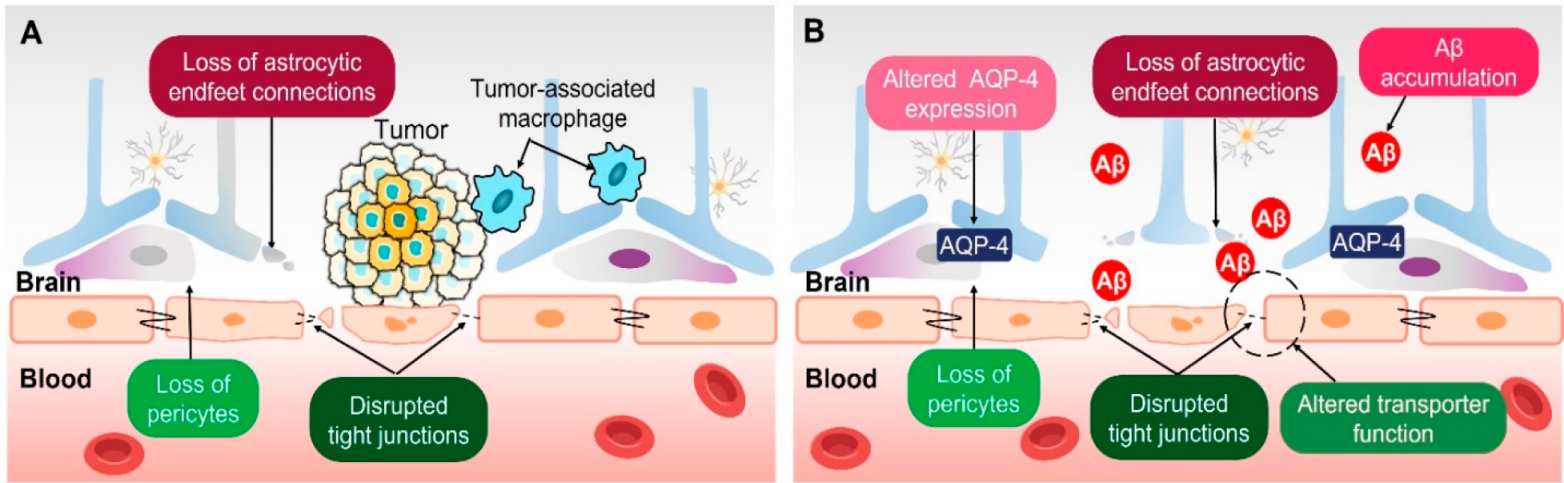
Changes of blood–brain barrier in brain tumors and Alzheimer’s disease. (A) Blood–brain tumor barrier (BBTB) characterized by abnormal and leaky blood vessels. Loss of tight junction proteins and astrocytic endfeet connections disrupts the integrity of the blood–brain barrier (BBB) and increases permeability. Reproduced with permission from ref (42). Copyright 2022 Wiley-Blackwell Publishing. (B) Changes in the blood–brain barrier (BBB) associated with Alzheimer’s disease (AD). AD-specific changes in the BBB involve disruption of tight junctions and changes in transporters in brain ECs, degradation of pericytes, and altered expression of aquaporin-4 (AQP4) in astrocytes, thereby resulting in enhanced permeability. Consequently, these alterations are closely related to the accumulation of amyloid-β (Aβ).

The blood–brain tumor barrier (BBTB) undergoes distinct structural and functional changes compared to the intact BBB in healthy brain tissue (Figure 4A).^40^ In glioblastoma (GBM), disruption of the BBB primarily occurs in the core of the tumor, where microvascular proliferation leads to newly formed leaky blood vessels. In contrast, the BBB tends to remain relatively intact in the peripheral zones surrounding the brain tumor.^41^ As the primary tumors proliferate, the neovascularization and intratumor blood vessels will deteriorate, causing damage to the BBB, whose structure and function become very different from those of the healthy BBB; this is called the BBTB. As a consequence, microvessels of the brain tumors are classified into continuous and nonfenestrated capillaries, continuous and fenestrated capillaries, and discontinuous (with or without fenestrations) capillaries based on their morphology. However, while often they are described as “leakier” than the BBB,^42^ some studies find the BBTB capable of expressing active efflux transporters and maintaining barrier integrity.^43^ Therefore, the leaky BBTB is highly heterogeneous with wide variability in permeation^44−46^ and abnormal tumor angiogenesis. This typically leads to a highly disorganized vasculature characterized by increased fenestrations and wider intercellular gaps that enhance paracellular transport. Tight junction proteins like claudin-3^47^ and occludin^48^ are frequently downregulated, further disrupting the barrier. Additionally, loss of pericytes, astrocyte endfeet connections,^49^ and neuronal connections^50^ could alter the BBTB microenvironment. BBTB permeability is also impacted by invading glioma cells that can physically displace astrocytic endfeet. Molecular transport changes are common, with upregulation of efflux pumps in the BBTB^51^ but also increases in endothelial pinocytic transport vesicles that facilitate transcellular drug uptake.^51^ Although the barrier still substantially limits delivery in many cases, these BBTB alterations result in enhanced penetration of chemotherapeutics, compared to that in the intact BBB. Increased immune cell infiltration is another characteristic of the BBTB change.^52^ The disorganized and heterogeneous BBTB structure promotes leakage of blood products, a phenomenon known as the enhanced permeability and retention (EPR) effect, which is typical in many cancerous tissues.^53^ Overall, understanding the distinct properties of BBTB presents opportunities to develop strategies to safely and effectively overcome this barrier and improve drug delivery to brain tumors.

### 3.3 BBB in Neurodegenerative Diseases

#### 3.3.1 BBB in Alzheimer’s Disease

AD is a prevalent neurological disorder, recognized as the primary cause of dementia.^54^ The initial occurrence associated with AD is the accumulation of Aβ, which can lead to cerebral amyloid angiopathy.^55^ The buildup of Aβ surrounding blood vessels has the potential to cause pericyte death^56^ and altered astrocyte morphology,^57^ consequently compromising BBB integrity (Figure 4B).^58^ Another major pathology hallmark in AD is the presence of tau deposits.^57^ Tau protein deposition also damages the vasculature, resulting in atrophic string capillaries and capillary surface irregularities.^59,60^ Additional BBB alterations in AD include endothelial abnormalities like mitochondrial damage, disrupted tight junctions, extracellular matrix alteration,^30^ reactive gliosis (early event in AD), molecular and functional changes in astrocytes, such as dysregulated potassium/calcium homeostasis and inflammation, reduced astrocyte metabolism (glycolysis and oxidative phosphorylation), astrocyte degeneration, increased aquaporin-4 expression in astrocytes,^61^ pericyte loss and reduced coverage on ECs, and peripheral immune cell infiltration.^62^ These widespread structural and functional changes to the neurovascular unit likely contribute to AD progression and cognitive decline.

#### 3.3.2 BBB in Parkinson’s Disease

Parkinson’s disease (PD) is characterized by the loss of dopaminergic neurons in the substantia nigra pars compacta and the presence of Lewy bodies, which are intracellular inclusions mainly formed by insoluble α-synuclein.^63^ The aging brain undergoes decreased capillary density, decreased blood flow, and impaired barrier integrity.^64^ In moderate-stage PD, an increase in vessel density indicates angiogenesis, while in late-stage disease, vessel density is significantly reduced, suggesting dynamic disease-stage-dependent vascular changes. Histological analyses have revealed capillary leakage, extravasated erythrocytes, perivascular hemosiderin deposits, and accumulation of serum proteins in PD patients.^65^ Activated microglia are mostly localized around blood vessels. PD-associated astrocytes are pro-inflammatory and fail to support capillary formation *in vitro*.^66^ Increased cerebrospinal fluid/serum ratios of albumin and IgG have also been reported in PD patients, indicating compromised BBB integrity.^67,68^ Several studies have demonstrated BBB disruption in PD using imaging approaches. For example, Bartels et al. found increased brain uptake of [^11^C]verapamil in PD patients, suggesting decreased P-glycoprotein efflux activity.^69^ Furthermore, Al-Bachari et al. showed greater contrast agent transfer across the BBB in PD patients compared to healthy controls, reflecting increased BBB permeability.^70^ Overall, substantial evidence points to BBB impairment as a critical pathophysiological component of PD.

## 4 BBB Transport Mechanisms

The physiological functions of the BBB involve the maintenance of ionic homeostasis and brain nutrition, regulation of neurotransmitter levels, and protection of the brain from exogenous neurotoxins and plasma macromolecules. We describe the transport mechanisms of the BBB under physiological conditions, and the endogenous and exogenous permeation mechanisms yielding NP crossing of the BBB.

**Figure 5:**
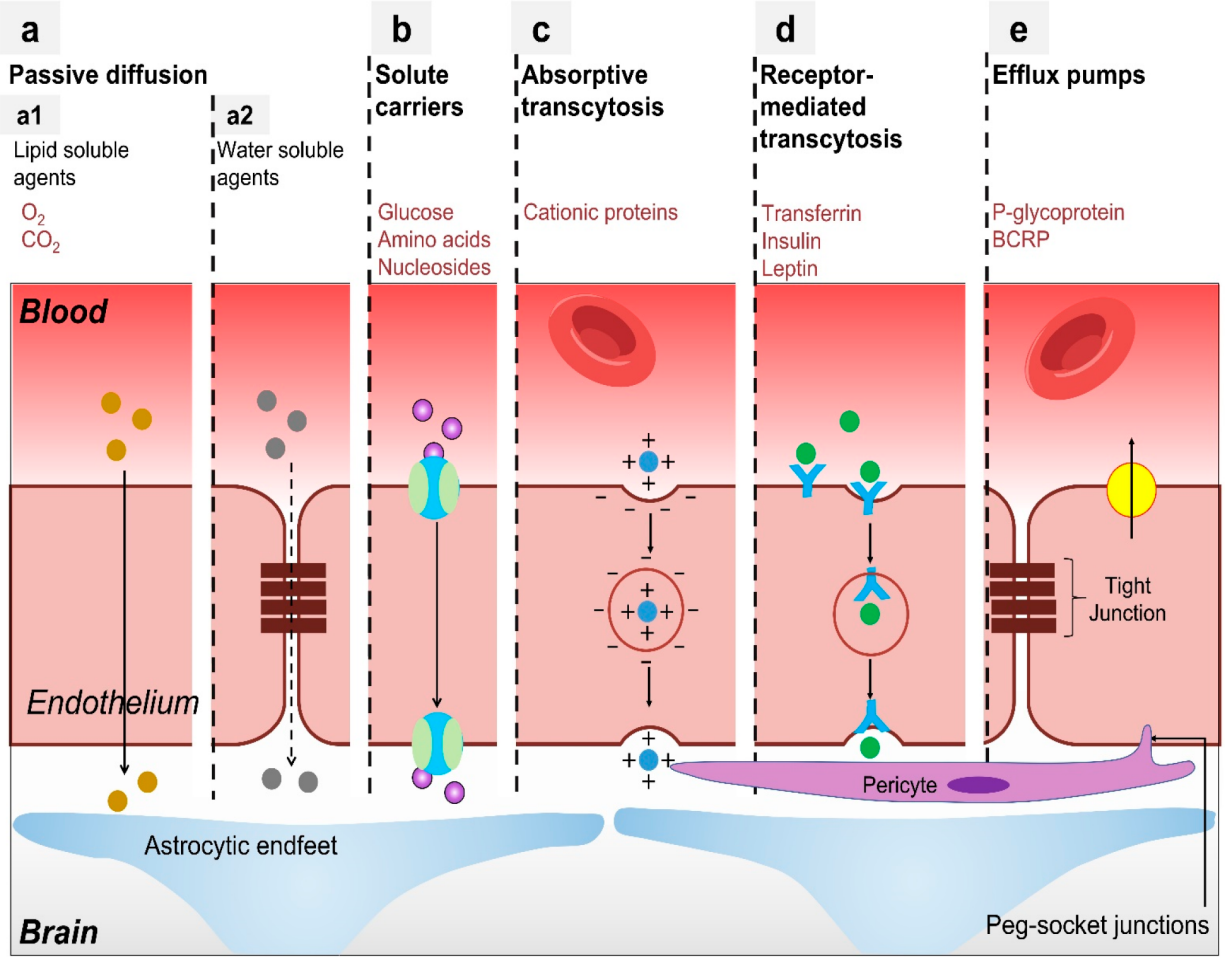
Physiological transport mechanisms crossing the BBB. Different transport pathways are presented, including (A) passive diffusion, driven by a concentration gradient, mainly involving (A1) transcellular pathways and (A2) paracellular pathways, (B) solute carrier, as occurs for glucose, amino acids, and nucleosides, (C) absorptive-mediated transcytosis for transport of cationic proteins in a nonspecific manner, (D) receptor-mediated transcytosis for the transport of transferrin, insulin, and leptin, and (E) efflux pumps (in the case of P-glycoprotein and BCRP). Reproduced with permission from ref (71). Copyright 2014 Walter De Gruyter.

Despite the highly restrictive transport of substances across the BBB, the brain has substantial demands for nutrients and energy. Therefore, multiple mechanisms mediate brain uptake of specific endogenous substances through passive or active transport routes. Generally, substances may cross the BBB under physiological conditions by (i) passive diffusion, (ii) carrier-mediated transport, (iii) adsorptive-mediated transcytosis, (iv) receptor-mediated transport, and (v) efflux pumps (Figure 5).^71^

### 4.1 Transport via Simple Diffusion

Passive transport is a nonspecific, energy-independent, concentration-dependent transport process that is limited to small molecules. In general, it can be categorized into paracellular and transcellular diffusion patterns. The presence of tight junctions (TJs) renders the intercellular space extremely narrow, thus physically hindering paracellular transport across the BBB. However, small lipophilic molecules, with a molecular weight <400–500 Da and a number of hydrogen bonds <8–10, may enter the brain through lipid-mediated free diffusion.^11^ In addition, small gaseous molecules such as O_2_ and CO_2_ rapidly and freely diffuse between the blood and the brain parenchyma.^21^

### 4.2 Carrier-Mediated Transport

Carrier-mediated transport is one of the most prevalent transport mechanisms across BBB, where nutrients, such as glucose, amino acids, and nucleosides, are transported.^72^ It can be classified as facilitated transportation or secondary active transport. The facilitated transportation does not require energy since it is driven by electrochemical or concentration gradients. However, secondary active transport means the transport of a solute in the direction of its increasing electrochemical potential coupled with facilitated diffusion. These solute carriers may be specific to one molecule or to several molecules. By this path, the nutrient molecule first binds to the specific transporter at the luminal side. Then the transporter changes its conformation to transfer it into the brain parenchyma.

### 4.3 Adsorptive-Mediated Transcytosis

Adsorptive-mediated transcytosis (AMT) is induced by electrostatic interactions between positively charged cationic molecules and negatively charged membrane surface domains,^73^ causing cell membrane invagination and vesicle formation primarily through negatively charged clathrin-coated pits and through caveolae.^74^ The EC membrane is covered by a glycocalyx, composed of heparan sulfate proteoglycans (HSPGs). HSPGs contain one or more covalently attached highly negatively charged heparan sulfate chains. Additionally, the sialoglycoproteins and sialoglycolipids contribute to a negative surface charge on the BBB. These factors work together to make the BBB luminal side highly negatively charged.^75^ Therefore, cationic molecules can be transferred into the brain parenchyma through interaction with the negatively charged surface of the cell membrane, such as cationic proteins.

### 4.4 Receptor-Mediated Transcytosis

Receptor-mediated transcytosis (RMT) is the main pathway to transport large molecules across the BBB. It is used to transport components like iron, insulin, and leptin in a homeostatic manner,^76^ as their receptors are highly expressed on the luminal surface of the BBB. It usually initiates with the binding of ligands with specific receptors on the luminal surface of the ECs, subsequently triggering membrane invagination and endocytosis. Intracellular transport vesicles involve either clathrin- or caveolae-mediated mechanisms.^77^ Clathrin-mediated endocytosis is characterized by the formation of “clathrin coat”, a polyhedral structure (clathrin triskelion). The adaptor protein AP2 initiates the clathrin assembly and the recruitment of other accessory proteins. Once completed, dynamin and other adaptor proteins separate clathrin-coated vesicles from the cell membrane through scission. Then, the clathrin coat will be rapidly removed and the internalized vesicle is free and primed to be trafficked and fused with an early endosome.^78^ This is the main transport mechanism of numerous endogenous substances, such as transferrin (Tf). Caveolae-mediated endocytosis and trafficking are also involved in the transport of lipids and fatty acids in ECs, by forming a small flask-shaped depression of plasma membrane, composed of phospholipids, sphingolipids, and cholesterol^79^ in the following steps: (1) budding and detachment from the cell membrane, (2a) fusion with endosomes followed by accumulation into lysosomes (classic), (2b) non-endosomal trafficking to intracellular organelles, or (2c) transporting substances from the bloodstream to tissues as a transcellular carrier (in endothelia), and (3) recycling of caveolae.^80^

### 4.5 Transport via Efflux Pumps

Efflux pumps are proteins localized on the endothelial membrane and use ATP hydrolysis to translocate solutes across the cellular membrane. Therefore, they can force the efflux of solutes against a concentration gradient. They are important machinery for expelling unwanted solutes into the capillary lumen. The principal efflux pumps in the BBB include P-glycoprotein (P-gp), breast cancer resistance protein (BCRP), and multidrug resistance-associated proteins.^81^ The inhibition of efflux pumps is another potential strategy for cargo delivery through the BBB and into the CNS parenchyma.

## 5 Permeation Mechanisms of Nanoparticles Capable of Crossing the BBB

NPs have been widely developed to deliver therapeutic agents through the BBB via endogenous or exogenous transport mechanisms. NPs can be functionalized to hijack the transport mechanism of endogenous substances (glucose and transferrin, Table 1) or cross through temporary BBB disruption exploiting exogenous effects (mannitol or focused ultrasound, Table 2).

**Table 1:** Nanoparticle System-Based Drug Delivery through Endogenous Transport Mechanisms

transport mechanism	uptake mechanism	size (nm)	zeta potential (mV)	target model of the BBB	ref
simple diffusion	diffusion	2.28 ± 0.32	a	spheroids	(85)
	direct passage by ion channels	2.5	a	rats	(86)
carrier mediated transcytosis	glucose transporter (GLUT)	118	a	APP/PS1 mice	(103)
		178.5 ± 2.3	15.5 ± 1.3	bEnd.l/3 cells and mice	(104)
		172.0 ± 3.7	13.5 ± 0.7		
	large neutral amino-acid transporter 1 (LAT-1)	89.6 ± 4.1	–34.6 ± 1.0	hCMEC/D3 cells and primary rat brain ECs	(105)
		385 ± 21	0.554 ± 0.110	rats	(106)
absorptive mediated transcytosis	electrostatic interaction	156 ± 10.85	22 ± 2.1	bEnd.3 cells	(107)
		82.1 ± 9.3	20.3 ± 3.7	hCMEC/D3 cells	(108)
		261 ± 8	45 ± 1	hCMEC/D3 cells	(109)
receptor mediated transcytosis	transferrin receptor (TfR)	6.612 ± 0.8893 (radius)	a	bEnd.3 cells, hCMEC/D3 cells, and glioma-bearing mice	(110)
	lactoferrin receptor (LfR)	a	a	human HBEC-5i cells	(111)
		a	a		
	low-density lipoprotein (LDL) receptor family	a	19	mouse brain ECs and APP/PS1 AD mice	(112)
		57.7–107.7	–3.6 (fresh)	bEnd.3 cells, mice and brain metastasis-bearing mice	(113)
	high-density lipoprotein receptor (HDLR)	a	a	HBMECs	(114)
	α_v_β_3_ integrin	192 ± 7	a	hCMEC/D3 cells, bEnd.3 cells, and glioma-bearing mice	(115)
efflux pump	P-gp	482.4 ± 33.0	–41.2 ± 2.7	hCMEC/D3 cells	(116)
	P-gp	∼105	–10.1	hCMEC/D3 cells	(117)

a Not applicable.

**Table 2:** Nanoparticle System-Based Drug Delivery through Exogenous Transport Mechanisms

interference agents	penetration mechanism	targeted disease	target model of BBB	ref
osmotic agents	osmotic opening	a	mice	(119)
focused ultrasound (FUS)	interaction of FUS with injected microbubbles	Alzheimer’s disease	APP/PS1 mice, BALB/c mice	(144)
laser	tight junctions (TJs)	a	mice	(130)
magnetic field force	influence of the magnetic field	a	HBMECs	(133)
	influence of the hyperpermeability of the tumor vasculature and magnetic field	glioma	glioma-bearing rats	(134)
cell-mediated transcytosis	response to brain inflammation	glioblastoma (GBM)	bEnd.3 cells and GBM bearing mice	(143)

a Not applicable.

### 5.1 Endogenous Transport Mechanisms of Nanoparticles Capable of Crossing the BBB

#### 5.1.1 Passive Diffusion

The TJs between ECs have a gap of 4–6 nm,^82^ which allows the particles smaller than 4 nm to cross the BBB through passive diffusion. First, small NPs like gold nanoparticles (AuNPs)^83^ and carbon dots (CDs) or lipid-based formulations may freely cross the BBB via passive diffusion. AuNPs are clustered particles with sizes ranging from 1 to 100 nm consisting of a gold core and a surface coating.^84^ When dispersed in a fluid, usually water, they are known as colloidal gold. Their small size allows them to cross biological barriers, e.g., the BBB. Sokolova et al.^85^ observed that the concentration of ∼2 nm core diameter AuNPs increased over time inside the spheroid (an *in vitro* BBB model) conceivably by the passive diffusion mechanism. Spheroids have been developed as *in vitro* BBB models, demonstrating typical restrictive permeability and comprising various brain cells including astrocytes, neurons, microglia, oligodendrocytes, pericytes, and a surface layer of endothelial cells. Using these spheroid models, time-dependent accumulation of AuNPs in the spheroid core was observed after 30 min, 6 h, and 24 h of incubation.^85^ Sela et al.^86^ synthesized 1.3 ± 0.3 nm AuNPs and found apparent accumulation in the frontal cortex, hippocampus, and hypothalamus of rats 6–16 h after intra-abdominal injection (200 μL, 105 mg/L). However, it was also noticed that AuNP crossing from capillaries into the brain parenchyma may be mediated by ion channels. Ca^2+^, Na^+^, and K^+^ channel blockers halved the concentration of AuNPs crossing the BBB compared to the corresponding value found in the control rats. This was explained by two possible mechanisms: (1) the blockers reduce the direct passage of AuNPs through the ion channels, where the diameters of Ca^2+^, Na^+^, and K^+^ are in the range of 0.9–1.5 nm, comparable to the size of the AuNPs, or (2) the blockers affect the ionic balance and lead to a reduction in BBB permeability through the TJs, therefore reducing the AuNP transfer.^86^

CDs are carbon-based core–shell NPs with low toxicity due to their lack of metals. They can penetrate the BBB by passive diffusion due to their small particle sizes (1–10 nm), low surface charge, and amphiphilicity.^87,88^ Zhou et al. found that CDs could cross the BBB due to their unique properties, including small size (3.4 ± 1.0 nm), low zeta potential (−15.3 mV), and amphiphilicity. CDs accumulated in the brains of zebrafish after 12 h of soaking, likely due to amphiphilicity.^89^ Carbon nitride dots under 2 nm also crossed the BBB through passive diffusion, as seen by their appearance in the central canal of zebrafish spinal cord 10 min after intravascular injection.^88^ Deng et al. showed that ∼2 nm amphiphilic CDs could cross the BBB with 10–20% efficiency *in vitro*. Furthermore, guanidine groups were introduced to CDs to interact with anionic TJs via cationic charge.^90^ Overall, these studies demonstrate that CDs are a promising NP platform for enhanced BBB permeation through passive diffusion. In addition, lipid-based carriers, such as solid lipid nanoparticles (SLNs) and nanostructured lipid carriers (NLCs), can penetrate across the BBB and be translocated in the brain through passive diffusion due to the lipid nature of the BBB^91,92^ and the nanometric size (40–200 nm).^93^ They are promising lipid-based formulations to treat CNS diseases. SLNs are prepared using solid lipids, such as mono-, di-, and triglycerides, fatty acids, waxes, and steroids. Adding various physiologically compatible emulsifiers, i.e., phospholipids, poloxamers, and polysorbates, also stabilizes NP formulations. Similarly, NLCs are modified SLNs in which the lipid phase consists of liquid lipids (oils) and solids at room temperature.^94^ The small size and lipid nature of SLNs and NLCs facilitate crossing the BBB, especially in the leaky tumor vasculature.^95,96^ Regarding drug delivery to the CNS, both SLNs and NLCs have been confirmed to improve the treatment of several neurological diseases, such as Parkinson’s disease,^97^ Alzheimer’s disease,^98^ migraine, epilepsy, and brain cancer.^92^ Many studies demonstrated the superiority of NLCs and SLNs over other nanoparticles (e.g., PLGA NPs^99^) to promote targeted delivery of drugs to the brain due to their lipid properties.^100−102^

#### 5.1.2 Carrier-Mediated Transport

The most explored carriers on ECs that are targeted for transporting NPs across the BBB are glucose transporters (GLUTs) and the l-type amino acid transporter (LAT). Zhou et al. reported that galactose-conjugated NPs can be delivered into APP/PS1 transgenic AD mice through the interaction with GLUT1 on ECs.^103^ Arora et al. found that dual modification with mannose (through GLUT1) and penetrating peptides in the liposomes can promote selectivity and enhance prospective delivery to the brain.^104^ Besides, LAT-1 is utilized as the target carrier for L-DOPA or l-valine conjugated NPs and enhances their entrance into the brain.^105,106^

#### 5.1.3 Adsorptive-Mediated Transcytosis

NPs could be functionalized with cationic components to enhance electrostatic interaction with the anionic surface of endothelial cells in order to facilitate their adsorptive-mediated transcytosis. Muniswamy et al. discovered that “dendrimer-cationized-albumin” encrusted NPs presented superior permeation transport across monolayer mouse brain endothelial bEnd.3 cells.^107^ Likewise, drug-loaded cationic liposomes (CLPs) could cross the immortalized human brain microvascular endothelial (hCMEC/D3) monolayer cells (*in vitro* BBB model).^108^ Chitosan-coated human serum albumin NPs can also interact with negatively charged cell membranes, subsequently crossing into hCMEC/D3 cells, accompanied by reversible and transient tight junction opening between the cells.^109^ Positively charged NPs offer superior efficacy of drugs in the CNS models while significantly improving BBB permeation. Hence, they could be ideal candidates for cargo delivery to the brain.

#### 5.1.4 Receptor-Mediated Transcytosis

Typically, for receptor-mediated transcytosis, NPs are conjugated with specific ligands. Fan et al. showed that H-ferritin (HFn) is an ideal ligand for conjugated-NPs to cross the intact BBB.^110^ Similarly, lactoferrin,^111^ angiopoietin-2,^112,113^ apolipoprotein A1,^114^ and cRGD^115^ were also conjugated with NPs to facilitate their penetration into the brain parenchyma. Ligand-modified NPs show excellent BBB crossing ability through the interaction with receptors expressed on healthy brain ECs. Interestingly, some ligand-modified NPs also show dual targeting abilities (BBB traversing and targeting the brain lesion) because the ligands on the NP surface can also bind to receptors expressed on abnormal brain cells. Therefore, these NPs may possess great potential to serve as an approach to BBB crossing as well as CNS selective treatments.

#### 5.1.5 Efflux Pump Inhibitors

The most explored efflux pump present on cerebral ECs is P-gp. Specific P-gp inhibitors have been used as a strategy for reducing the efflux of NPs and enhancing their entry into the brain. For instance, Gomes et al. revealed that reducing P-gp expression by siRNA NPs could enhance P-gp substrate permeability via modulating drug efflux at the BBB.^116^ Similarly, PEGylated PLGA NPs were applied to transport coumarin C75 for the treatment of PD, most probably via the inhibition of the P-gp efflux pump in hCMEC/D3 cells.^117^ The BBB-targeted NP induced P-gp down-regulation and consequently increased P-gp substrate permeability across brain membranes would allow for successful influx of drugs across the BBB.

In conclusion, passive diffusion can only be employed in some ultrasmall NPs, usually with a size of less than 4 nm. Their surface might also be modified to enhance the BBB crossing through specific modification methods, e.g., positive charge, ligands, or antibodies.^82^ Other passive and active mechanisms could also be responsible for the active transportation of molecules. Indeed, some of these mechanisms are exploited for the delivery of NP-conjugated drug(s) into the brain through the “Trojan horse” strategy. Their conjugation with targeting moieties can directly target brain endothelium and facilitate the internalization of cargos via endocytosis or transcytosis. Among these, (i) carrier-mediated transporters, (ii) adsorptive-mediated endocytosis, (iii) receptor-mediated endocytosis, and (iv) active efflux transporters are the most explored models for NP delivery in treating brain diseases.

### 5.2 Exogenous Transport Mechanisms of Nanoparticles Capable of Crossing the BBB

#### 5.2.1 Osmotic Agents

In addition to passive and active transport mechanisms, other forms of BBB manipulations could enhance its permeability, which in turn would increase the possibility of NP crossing. An example of such manipulation is using osmotic agents such as mannitol, ethanol, and dimethyl sulfoxide (DMSO). These agents could facilitate, increase, and improve the transport of different medicines into the brain tissue. Mannitol, which is used to reduce high intraocular pressure and intracranial hypertension,^118^ is an osmotic diuretic and metabolically inert in humans. It is often infused via an intra-arterial route to temporarily manipulate the permeability of the BBB^119^ and to enable passage of NPs with their cargos from blood vessel capillaries to the brain parenchyma.^120^ It was shown that BBB opening with mannitol resulted in faster and higher accumulation of ^89^Zr-bevacizumab deferoxamine, and interestingly, this was observed only in the ipsilateral hemisphere.^121^

#### 5.2.2 Focused Ultrasound (FUS)

Focused ultrasound (FUS) is a noninvasive technique for targeted and reversible BBB interruption with sub-millimeter accuracy that shows significant potential for improving drug delivery into the brain parenchyma and treatment of CNS diseases.^122^ Transcranial FUS is frequently combined with intravenously delivered microbubbles (MBs) to disrupt the BBB membrane physical integrity.^123,124^ Briefly, the MBs steadily oscillate in a limited and specific area within an acoustic field to create mechanical shear forces and circumferential stresses on the BBB microvasculature walls.^125,126^ This would transiently open the BBB in a spatially targeted manner, though the entire structural integrity of the BBB will be restored within 4–6 h after the treatment.^127^ In a recent phase I clinical trial, Gasca-Salas et al. (NCT03608553) investigated the efficacy of MR-guided focused ultrasound combined with intravenous microbubbles for temporarily opening the BBB in patients with Parkinson’s disease and dementia. The treatments successfully achieved BBB opening in the parieto-occipito-temporal junction in 8 out of 10 treatments across 5 patients, as evidenced by gadolinium enhancement on MRI.^128^ Furthermore, the MRI results revealed BBB closure in the putamen shortly after treatment. Importantly, no hemorrhagic or infarct lesions were detected.^129^ These promising findings demonstrate the safety and effectiveness of combining focused ultrasound with microbubbles for temporary BBB opening.

#### 5.2.3 Laser

Laser stimulation has also been investigated to increase BBB paracellular diffusion of NPs by enhancing permeability through the TJs.^130,131^ The local bioeffects generated by the interactions between NPs and picosecond-laser pulses induce a temporary BBB permeability and a higher payload accumulation via diffusion through the paracellular tight junction. Li et al.^130^ labeled blood vessels with lectins and then perfused animals to remove excess liposomes from the blood vessels and demonstrated that the BBB manipulation via laser allows antibodies, genes, and liposomes to penetrate the brain parenchyma. This practically presented a valuable, safe, and effective medicinal delivery method in the CNS.^130^

#### 5.2.4 Magnetic Field Force

Magnetic nanoparticles (MNPs) are composed of a magnetic iron oxide core and biocompatible surface (e.g., dextran, lipids, polymers, or small molecules). The core is most often magnetite (Fe_3_O_4_) or maghemite (α- or γ-Fe_2_O_3_) with sizes of 1–100 nm.^132^ They have been used in drug delivery to the brain. Studies with magnetic NPs are mainly established based on the following three mechanisms: (1) modification with functional ligands, (2) application of an external magnet field (EMF), and (3) use of a low radiofrequency field. The first strategy is similar to RMT, which utilizes peptides, antibodies, and small molecules as NP-attached ligands to facilitate their transport to the brain. The second strategy is to use external magnet force to move MNPs where they can be directed from the blood vessel lumen to the brain parenchyma.^133,134^ Magnetic forces can temporarily disrupt endothelial cell–cell junctions through internalized MNPs, activating the paracellular transport pathway and facilitating the local extravasation of circulating substances.^135^ The penetration efficiency is closely related to MNP properties (size, magnetic properties, coating, functionalization, and biocompatibility) and external factors (the strength, gradient, and geometry of the magnetic field, the blood viscosity, and flux velocity).^136^ Chen et al. found that a static magnetic field assisted MNP penetration through the BBB, increasing it to 8.47% compared to 3.36% without magnetic field treatment.^137^ Gupta et al. found that MNPs could penetrate the BBB through the transient disruption of ZO-1, an indicator of BBB integrity. ZO-1 expression was reduced 1 h after magnetic field exposure, partially recovered at 2 h, and reached comparable levels to normal at 3 h. These results validate that MNPs can transiently open TJs to cross the BBB through magnetic hyperthermia induced by the magnetic field. The combination of an alternating magnetic field and an external static magnetic field enhanced MNP crossing to ∼63%, compared to ∼50% with just the external static magnetic field alone. Besides, more MNP accumulation in the mouse brain was observed when applying an alternating magnetic field, demonstrating its ability to modulate BBB permeability through the magnetic guidance of NPs.^138^ Gkountas et al.^139^ demonstrated that an EMF could significantly enhance MNP delivery across the BBB. Without EMF exposure, 100 nm MNPs showed minimal BBB penetration (2–6%). However, under an EMF intensity of 0.39 T, the BBB crossing efficiency of 100 nm MNPs markedly rose to 40%. Meanwhile, the percentage of 10 nm MNPs passing the BBB was unchanged (<1% difference) with or without EMF. In contrast, the permeability of larger 100 nm MNPs improved by up to 30% under EMF conditions. Maximum permeation of ∼45% was attained using an EMF intensity of 1 T, compared to 42.5% at 0.8 T, 37.5% at 0.5 T, and only 12.5% without EMF. Furthermore, manipulating fluid flow velocity also impacted MNP transcytosis. At a baseline flow of 10^–3^ m/s, the percentage of NPs passing the BBB reached 45%, decreasing to 32% under slow flow (5 × 10^–4^ m/s) but improving slightly to 48% with faster flow (2 × 10^–3^ m/s). This demonstrates that EMF intensity and fluid dynamics modulate MNP delivery across the BBB, providing insights into enhancing brain nanoparticle permeation via magnetic guidance.^139^ The third strategy is to utilize the heat produced by Néel relaxation of magnetic NPs under the radiofrequency field, which can also transiently and locally open the BBB.^2,140^

#### 5.2.5 Cell-Mediated Transcytosis

Cell-mediated transcytosis is a typical method for immune cells to cross the BBB. Different blood-borne immune cell populations, including leukocytes, monocytes, and macrophages, undergo diapedesis and cross the barrier via both paracellular and transcellular routes.^141^ This happens in the case of both autoimmune reaction and infection of the CNS.^142^ These cells cross the BBB, where there is an increased expression of adhesion molecules on the ECs. The discovery of this mechanism has explored for delivering therapeutic materials to the CNS and an example is the treatment of glioblastoma (GBM). Recently, Li et al. (2021) demonstrated that encapsulating NPs within neutrophils could significantly enhance drug delivery across the BBB *in vitro* and *in vivo*. They found that the penetration rate of the drug-loaded NPs across the barrier increased from 5.2% without encapsulation to 35.6% when enveloped within neutrophils. This effect was mediated by inflammation from GBM, which served to activate and attract the NP-containing neutrophils. In mice with glioblastoma, intravenous injection of the neutrophil-encapsulated NPs led to their accumulation in the brain tumor, with NP levels remaining significantly elevated for 48 h postinjection. This neutrophil “Trojan horse” approach could provide an effective cell-mediated transcytosis strategy to enhance NP drug delivery across the BBB for therapy.^143^

Various strategies have been employed to enable NP penetration across the BBB, including osmotic disruption, focused ultrasound, laser, magnetic guidance, and cell-mediated transcytosis. The former four techniques work primarily through the temporary disruption of TJs between brain ECs. In contrast, cell-mediated transcytosis takes advantage of innate immune cell trafficking pathways that are activated during neuroinflammation and in brain tumors like glioma. While showing promise in enhancing NP delivery to the brain, these strategies need to be further optimized to provide greater permeation while minimizing adverse effects like toxicity, damage to brain vasculature, and loss of BBB integrity.

## 6 Properties Affecting Nanoparticle Crossing through the BBB

Different strategies have been employed to improve the BBB penetration proficiency of NPs. Among various approaches,^145−148^ a common way is to regulate the Wnt/β-catenin signaling produced by glial cells and reduce their binding to multiprotein receptor complexes on brain ECs^23^ for cargo administration with high BBB penetration potential. The main limitation of all these methods, however, could be the structural damage and, hence, the dysfunction of the BBB.

**Figure 6:**
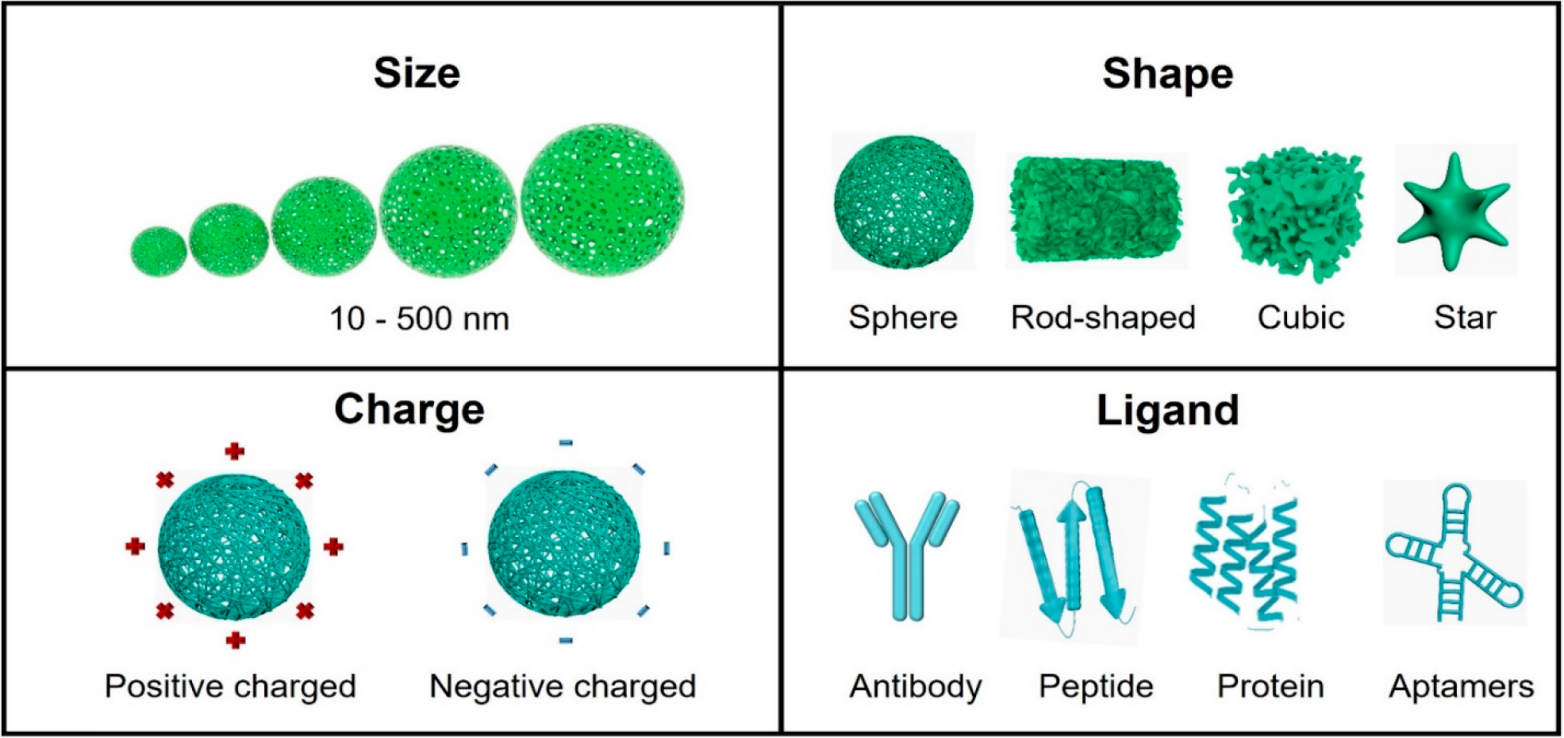
Overview of nanoparticles developed for the purpose of BBB penetration and their tunable parameters: size, shape, charge, and ligand.

Another effective strategy to improve the BBB penetration proficiency of NPs is to synthesize the NPs in such a manner that they could actively cross the BBB themselves. Here, we discuss the effects of size, shape, surface charge, and surface ligands of NPs on their BBB permeability (Figure 6).

### 6.1 Size Effect of Nanoparticles

Size effect is an essential factor that needs to be considered when designing NPs because it will directly affect their uptake and permeability through the BBB. NPs of different sizes show diverse circulation half-life time, vascular permeability, and macrophage clearance^149^ and have different tendencies to accumulate in different organs. For instance, NPs smaller than 5 nm in diameter could cross the BBB effortlessly. However, their short circulation time and rapid clearance by the kidney will limit their effectiveness and practical applications.^150^ NPs in the 5–15 nm size range exhibit broader organ distribution profiles, and particles smaller than 10 nm are prone to renal clearance. In particular, 15 nm NPs have been shown to achieve the highest distribution to the liver, lungs, and kidneys at 24 h postadministration, with additional biodistribution in the spleen, heart, brain, blood, and stomach. Meanwhile, 20 and 50 nm NPs exhibit maximal accumulation in the liver and spleen at 24 h, distributing additionally to blood, kidneys, heart, lungs, and cerebral cortex.^151,152^ On the other hand, NPs in the 50–100 nm size range tend to accumulate nonspecifically in the liver through fenestrations in sinusoidal endothelium.^153,154^ At 100–200 nm size, NPs undergo rapid clearance by macrophage phagocytosis in the liver and spleen.^155^ NPs at 200–500 nm are inclined to be retained in the spleen.^156^ Larger NPs of 2–5 μm size would accumulate in the pulmonary capillaries^157^ and then be cleared by the mononuclear phagocyte system. Obviously, the nanomaterial size, structure, and design are the most critical properties of NPs as carriers for the delivery of cargos across the BBB.

Betzer and colleagues^158^ investigated the effect of size on BBB permeability of insulin-coated gold NPs (INS-GNPs) in an *in vivo* model. They synthesized different sized INS-GNPs (20, 50, and 70 nm) and illustrated their distribution by CT and atomic spectroscopy after intravenous (iv) injection. The results showed that the intracerebral concentration of 20 nm INS-GNPs was higher than that of 50 and 70 nm INS-GNPs at 2 and 24 h after iv injection. This could be explained by the fact that the small sized INS-GNPs could cross the BBB easily.^158^ Cai et al.^159^ chose 2,3-bis(4-(phenyl(4-(1,2,2-triphenylvinyl)phenyl)amino)phenyl)-fumaronitrile (TPETPAFN) as an aggregation-induced emission fluorogen, which was encapsulated with PEG to form NPs of different sizes (10, 30, 60 nm). In their investigation, NPs of most sizes were unable to cross the intact BBB, but the NPs with smaller sizes (10 and 30 nm) were able to penetrate an altered BBB (induced by photothrombotic ischemia (PTI)).^159^ Although most of the NPs display size-dependent BBB permeability, obviously, this is not an absolute rule. Nowak et al.^160^ tested the BBB permeability of carboxylated polystyrene (PS) particles of different sizes (100, 200, and 500 nm) in an *in vitro* model. Their BBB replica model was established by immortalized human brain microvascular endothelial cells (hCMEC/D3). The PS particles at 200 nm showed better BBB penetration than the others (10-fold higher than 100 nm and 100-fold higher than 500 nm), indicating a non-monotonic dependence on their properties. This could be explained by the contact areas and the adhesion between NPs and ECs increasing with their size, but the sizes larger than 200 nm tend to accumulate in the kidney and, hence, lead to lower accumulation in the brain.^160^

### 6.2 Shape Effect of Nanoparticles

Shape is another key factor affecting the pharmacokinetics and BBB permeability of the NPs. NPs can be synthesized as spheres, rods, cubes, flat shapes, stars, etc. Their shape will affect their potential for targeting as well as BBB penetration due to their differences in contact quality and contact areas with the target cells in the BBB.^161^

Most nanocarriers are designed to be spherical because of their ease of manufacturing. Nevertheless, this has limited their biological properties, circulation time, targeting potential, and BBB penetration, because of their relatively lower contact areas.^154^ Da Silva-Candal and co-workers^162^ studied the binding affinity to the cerebral microvasculature cells of polystyrene NPs in diverse shapes (spherical and rod-shaped), in order to evaluate the effect of NP shape on BBB permeability. The binding ability of polystyrene NPs with cerebral microvasculature cells was tested in static (cell culture dishes coated with bEnd.3 cells) and dynamic (flow in microfluidic channels lined with hCMEC/D3 cells) conditions. The rod-shaped polystyrene NPs showed stronger binding ability (2.5-fold increase in static, 1.5-fold increase in dynamic) with cerebral microvasculature cells than the spherical NPs *in vitro*, because the contact areas between the rod-shaped NPs and the cells were much larger.^162^ Fu et al.^163^ found that the cellular endocytosis efficiency of PEGylated upconversion nanoparticles (UCNPs) was closely related to particle shape, as defined by various aspect ratios (length/width). UCNPs with an aspect ratio of 2 exhibited the highest uptake (86.8%) in hCMEC/D3 endothelial cells, compared to aspect ratios of 1 (spherical, 47.4% uptake), 3 (rod-shaped, 55.9% uptake), or 4 (rod-shaped, 37.6% uptake). UCNPs with aspect ratios of 3 or 4 displayed substantial aggregation and accumulation on cell surfaces, and lower uptake efficiency. In contrast, UCNPs with an aspect ratio of 2 showed good dispersibility and high accumulation in the brain of living zebrafish compared to UCNPs of other aspect ratios. Following UCNP injection in zebrafish, confocal microscopy with 980 nm laser excitation at 2 h postinjection revealed poor dispersion of UCNPs with high aspect ratios (3 or 4) from the injection site, correlating with the aggregation observed in cell cultures. In comparison, PEG-UCNPs with an aspect ratio of 2 exhibited diffusion from the injection site into the zebrafish brain. PEG-UCNPs with an aspect ratio of 1 also showed some diffusion into the brain, although less than those with aspect ratio 2. The brain uptake efficiency of aspect ratio 1 UCNPs was 33.5 ± 4.8% of that for aspect ratio 2. UCNPs with aspect ratio 3 had approximately half the brain uptake of those with aspect ratio 1, while those with aspect ratio 4 had the lowest uptake in the zebrafish brain (only 8.7 ± 0.6% of those with aspect ratio 2).^163^ Undoubtedly, the paradigm regarding NPs with different morphologies is of great significance to the delivery design concept in bio-nanotechnology tools and their application in nanomedicines.

### 6.3 Surface Charge Effect of Nanoparticles

The charge on the surface of polymeric NPs affects their properties, like circulation life, BBB permeability, cytotoxicity, and so on. Positively charged particles can effectively target tumor blood vessels, and neutrally charged particles show faster diffusion.^164^ Neutrally and positively charged NPs show lower serum protein adsorption in blood circulation, thus manifesting a longer circulating time than negatively charged NPs.^165^ Brain EC membranes contain relatively more negatively charged phosphatidylserine and phosphatidylinositol and exhibit higher negative charges than other EC vasculars. The high negative charge not only provides an additional protective barrier for the brain but also regulates the entry of macromolecules and other charged cargos, like drugs or carriers such as NPs, into the CNS.^166^ Cationic NPs can be adsorbed to the plasma membrane surface of the EC through electrostatic interaction and then cross the BBB by adsorptive-mediated endocytosis, thus, exhibiting higher BBB permeability.^167^ However, compared to neutral and negatively charged NPs, cationic NPs showed higher cytotoxicity to ECs, and high macrophage uptake and clearance.^168^ Therefore, it is important to consider the benefit-to-risk ratio and adjust the surface charge of NPs to make them highly penetrable to the BBB with low cytotoxicity. Zhang et al. investigated the BBB penetration of two different charged (neutral and positive) polystyrene nanospheres in an *in vitro* BBB model. Their results indicated that the positively charged NPs exhibited significantly higher BBB penetration capacity than the neutral molecules by approximately 100-fold.^169^ Chen et al.^170^ have demonstrated that the surface charge of mesoporous silica nanoparticles (MSNs) plays a critical role in their ability to cross the BBB in zebrafish models. Specifically, 50 nm MSNs with a highly negative surface charge (zeta potential of approximately −40 mV) achieved significant accumulation in the brain parenchyma of zebrafish embryos, superior to MSNs with lesser negative charge. In contrast, positively charged MSNs (+18.1 mV and +42.3 mV) showed very limited brain penetrance, indicating enhanced BBB crossing by negatively charged particles. Analysis of the NP protein corona revealed enrichment of three known BBB transporter proteins, afamin, apolipoprotein E, and basigin, on the highly anionic MSNs, providing a mechanistic rationale for their enhanced BBB penetrance compared to cationic and lower charged MSNs. Fluorescence microscopy of larval zebrafish brains demonstrated striking accumulation of N4-RMSN50@PEG/THPMP, a highly negative MSN variant, outside of blood vessels, reflecting extensive parenchymal diffusion. Far fewer cationic MSNs (P1- and P4-RMSN50@PEG/TMAC) or weakly negative particles (N1-RMSN50@PEG) crossed the BBB. High resolution two-photon microscopy and histological analysis corroborated and extended these findings, clearly delineating accumulation of N4-RMSN50@PEG/THPMP within the brain. Taken together, these results demonstrate that a highly negative nanoparticle surface charge promotes BBB transport likely by enrichment of protein corona components that engage endogenous BBB transport mechanisms. Modulation of surface charge could enhance the delivery of nanomedicines across the BBB and into the central nervous system.^170^

### 6.4 Surface Ligand Effect on Nanoparticles

In order to enhance the BBB permeability of NPs by receptor-mediated transcytosis (RMT), numerous promising targeting ligands such as plasma proteins, antibodies, peptides, and aptamers have been reported. For instance, transferrin (Tf) is ferritin that can cross the BBB by binding to the transferrin receptor (TfR) on the surface of ECs and has been widely used in the modification of nanocarriers as brain-targeting ligands.^171^ Kuo et al. developed solid lipid NPs grafted with Tf and folic acid (FA) to deliver genes (BV6 and GDC0152) into the brain for the treatment of GBM multiform. Such modification with the Tf could prolong the circulation time of the NPs by avoiding their direct contact with plasma proteins. Additionally, the BBB permeability was favored by Tf binding with the Tf receptor and FA receptor through RMT.^172^ Apolipoprotein E (ApoE) is a fat-binding protein, showing a high affinity to the low-density lipoprotein receptor (LDLR) and some low-density lipoprotein-associated receptors (LRPs). ApoE has been widely used for the functionalization of polymeric NPs to elevate their BBB permeability.^173^ Zhang et al. (2020) constructed NPs with bovine serum albumin (BSA) coated with a dopamine shell, then linked COG1410 (an apolipoprotein E mimetic peptide that can transport NPs across the BBB) on the surface. This nanoplatform could effectively deliver itraconazole into the brain, and display promising therapeutic effects for meningitis caused by *Candida albicans*.^174^ Moreover, platelet-derived growth factor receptor β (PDGFRβ) is a kind of tyrosine kinase receptor located on the surface of the cell membrane. Its function mainly is to regulate cell proliferation, differentiation, growth, and development. Monaco et al.^175^ developed a nanovector conjugated with the anti-PDGFRβ aptamer. This nanovector was successfully used to deliver the chemotherapeutic drug NVP-BEZ235 into the brain and target the PDGFRβ overexpressed on the endothelial cells of vessels. Furthermore, the anti-PDGFRβ nanovectors accumulated in the brain at 2 and 4 h after systemic administration.^175^ Li et al.^176^ demonstrated that modification of NPs with the rabies virus glycoprotein peptide (RVG29) enabled effective transport across the BBB in cellular and animal models of Parkinson’s disease. In bEnd.3 BBB model cultures, RVG29-functionalized NPs encapsulated in neutrophil-derived membranes (RVG@AHM@Pt/CeO2) exhibited a 2.12-fold enhancement in BBB transit compared to nontargeted particles (AHM@Pt/CeO2). This was accompanied by less retention of RVG-modified NPs (81.33% of that in AHM@Pt/CeO2) in the upper chamber after transit, reflecting excellent BBB penetration performance. Similarly, *in vivo* imaging in Parkinson’s mice showed pronounced BBB permeability and brain retention only for RVG29-modified, cell membrane coated NPs. Those NPs lacking RVG29 functionalization remained largely within the circulation. Together, these findings demonstrate that rational modification of cell membrane-coated NPs can achieve targeted delivery across the BBB. The modular RVG29 targeting ligand, derived from a neurotropic virus, binds specifically to receptors at the BBB to trigger transcytosis. Combining cell membrane camouflage and selective BBB targeting ligands is a promising approach to improving central nervous system delivery of NPs for therapy development.^176^ Zhang et al.^177^ demonstrated a micelle-based drug delivery system that enhanced the transport of antiepileptic drugs (AEDs) across the BBB. The micelles were comprised of ferrocene-conjugated d-α-tocopherol poly(ethylene glycol succinate) (TPGS-Fc) and amphiphilic copolymer Poloxamer 407, enabling high encapsulation of diverse AEDs. Key features of the TPGS-Fc micelles, including transferrin receptor binding and P-glycoprotein efflux pump inhibition, promoted BBB penetration. Cellular uptake and BBB transmigration studies in bEnd.3 models verified improved BBB transit of TPGS-Fc micelles loaded with a fluorescent probe compared to free drug. They showed highly enhanced fluorescence intensity (31.5 ± 8.2%), which demonstrated the high efficiency in improving drug penetration of the BBB. BBB transit was decreased from 28.7 ± 3.4% to 18.9 ± 3.1% through pretreatment with transferrin, implicating receptor-mediated transcytosis as the mechanism. ATP assays also revealed that TPGS-Fc micelles inhibit P-glycoprotein, further enhancing brain permeation by averting efflux. *In vivo* imaging in mice showed a 2.4-fold increase in brain accumulation of AEDs loaded in TPGS-Fc micelles compared to free dye at 36 h postinjection. Whole brain imaging and sectioning corroborated micelle-mediated enhancement of brain delivery, 2.4-fold that in the free dye group. In summary, the rational design of TPGS-Fc micelles promoted the delivery of AEDs across the BBB via transferrin receptor binding and P-glycoprotein inhibition. This system shows promise for improved pharmacotherapy of epilepsy and potentially other CNS disorders.^177^ In another study, Nong et al.^178^ demonstrated that targeting the adhesion molecule ICAM-1 enabled the transport of NPs across the BBB. They intravenously injected NPs conjugated to anti-ICAM-1 antibodies. Initially, a large fraction (>20%) of the anti-ICAM NPs localized to the lungs and associated with pulmonary leukocytes (>90%). Over time of 0.5 h, 4 h, and 22 h, the anti-ICAM NPs and pan-leukocyte marker (CD45) decreased in the lungs and increased up to 5-fold in the brain, suggesting the initial delivery of αICAM to activated leukocytes in the pulmonary vasculature followed by migration of leukocyte-loaded NPs to the injured brain across the BBB. Intravital microscopy visually confirmed anti-ICAM nanoparticles localized to inflamed brain vasculature after injection. The number of detectable NPs in the brain parenchyma increased over 22 h, indicating the crossing of the BBB. Flow cytometry revealed the nanoparticles in the brain were almost entirely taken up by leukocytes (98.7%). Specifically, 73% of nanoparticle-positive leukocytes were monocytes/macrophages and 24.5% were neutrophils. Histology showed nanoparticles largely colocalized with macrophages in the brain parenchyma. In summary, targeting ICAM-1 enables leukocytes to act as Trojan horses to carry the NPs across the BBB during neuroinflammation.^178^

## 7 Functionalized Polymeric Nanomaterials Crossing the BBB

Many nanomaterials, encompassing poly(ethylene glycol), poly(lactic-*co*-glycolic acid) and other categories, exhibit distinctive physicochemical properties and multifunctional modification, rendering them pivotal carriers for therapeutic agents across the BBB. The exploration of various nanoparticles, including those based on polymers, lipids, and metals, has been a focal point of research over the years, leading to the emergence of noteworthy findings (Table 3).

**Table 3:** Summary of Various Polymeric NPs Capable of Crossing the BBB

polymer	BBB targeting agents	application	ref
PEG	Pen peptide	AD therapy	(179)
PEG	AGBBB015F and Regulon peptides	brain glioma treatment	(180)
PEG	lactoferrin	glioblastoma therapy	(181)
PEG	lactoferrin	BBB crossing	(182)
PEG	CD4^+^ T_EM_ cells	BBB crossing	(183)
PLGA	DBP	AD therapy	(184)
PLGA	RVG29 peptide	BBB crossing	(185)
PLGA	Angiopep-2 peptide	BBB crossing	(187)
PLGA–PEG	RVG29 peptide	PD treatment	(188)
liposomes	RVG29 peptide	PD treatment	(189)
PEGylated liposomes	transferrin	glioblastoma therapy	(191)
lipoprotein	T7 peptide	glioma-targeted therapy	(192)
PEI	RGD peptide	glioblastoma therapy	(193)
PEI	cRGD peptide	glioblastoma therapy	(194)
PEI–PEG	HER2 antibody	malignant glioma treatment	(195)
PLA	MPC monomer	BBB crossing	(197)

### 7.1 Poly(ethylene glycol) (PEG)-Modified Nanomaterials

**Figure 7:**
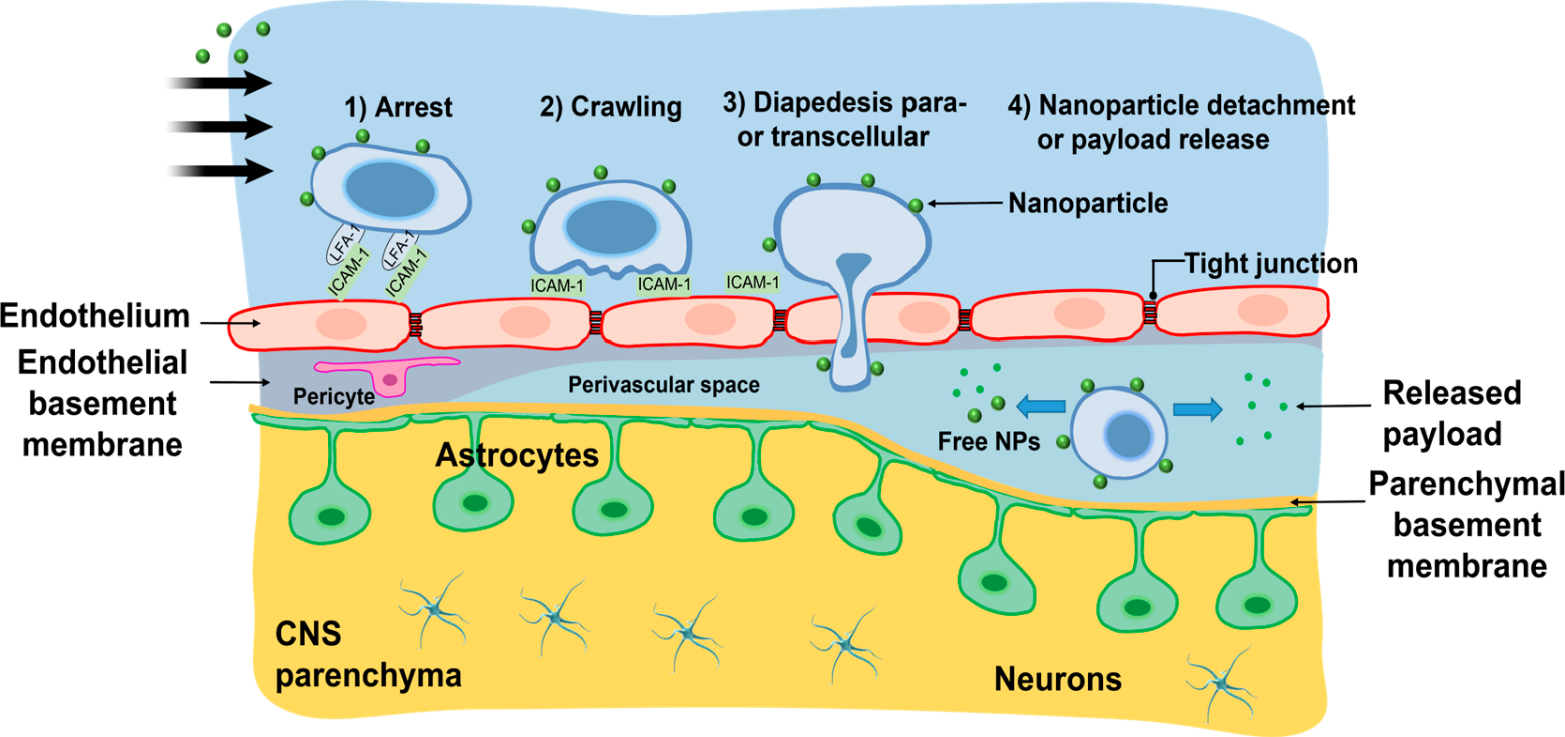
Schematic diagram of NP transport over the BBB via CD4^+^ TEM cells. After reaching the CNS parenchyma, the NPs first attach to the myeloid cells and penetrate the BBB endothelium before detaching themselves and releasing their cargos. Reproduced with permission under a Creative Commons License from ref (183). Copyright 2021 the Authors, published by Wiley-VCH GmbH).

Poly(ethylene glycol) (PEG) is commonly manufactured by the addition polymerization of ethylene oxide and water or ethylene glycol with the chemical formula HO(CH_2_CH_2_O)_*n*_H. PEG is widely employed in biomedical applications because of its outstanding biocompatibility in physiological microenvironments. For example, Ru@Pen@PEG-AuNS was constructed by Yin et al.^179^ to alleviate AD, in which these NPs specifically acted as nanovehicles with higher BBB permeability potential. In their study, the gold nanostars (AuNSs) were formed via a seed-mediated growth process and served as photothermal agents under 808 nm laser excitation. The biocompatibility and physiological stability of the NPs were also significantly improved through linking with PEG. The surface of PEG-AuNS was also coated with the penetratin (Pen) peptide, a cell-penetrating peptide (CPP) of 16 amino acid residues that has a strong ability for cellular internalization and, hence, BBB crossing. Lastly, the final NPs were tagged with ruthenium (Ru) to obtain the luminescent attribute. The final Ru@Pen@PEG-AuNS NP construct demonstrated a strong neuroprotective benefit and great BBB diffusion in the AD mouse model.^179^ In 2018, polyester-based NPs with biodegradable block copolymers were prepared by Borrós et al.^180^ through the polycondensation technique. These NPs (i) coated with PEG, (ii) conjugated with BBB-crossing peptides (AGBBB015F and Regulon REG), and (iii) loaded with PTX were effectively employed to treat glioma.^180^ Overall, such NPs with high drug loading capacity (by exhibiting a strong binding affinity with low-density lipoprotein receptors) could facilitate crossing of the BBB via receptor-mediated transcytosis. Additionally, Kuo et al. (2016) developed a form of BBB-crossing solid lipid-based nanocarriers (SLNs) designed with tamoxifen (TX) and lactoferrin (Lf) ligands to deliver carmustine (BCNU), which is an anticancer agent and GBM multiforme growth suppressor, into the brain parenchyma.^181^ Interestingly, the BCNU-loaded SLNs that have been customized with TX and Lf ligands displayed ten times higher BBB permeability compared to the naked BCNU-loaded SLNs. In this dynamic system, the Lf ligand was exploited to coordinate the transcytosis across the BBB through a receptor-mediated mechanism, and the TX acted as a selective estrogen receptor modulator to compensate for the efflux loss. These NPs have been acknowledged as promising delivery agents for GBM pharmacotherapy with noticeable properties in crossing the BBB.^181^ Furthermore, Song and co-workers^182^ designed a pharmaceutical brain-delivery system through a microemulsion process to fabricate biocompatible silica NPs and covalently modified them with Lf receptor and PEG. The Lf facilitated BBB targeting by the receptor-mediated transcytosis process. In addition, these NPs could avoid being trapped in the reticuloendothelial system by PEG due to their substantial solubility and long half-life in blood circulation. The size of these specially designed NPs ranged from 25 to 100 nm. Obviously, the smaller size NPs showed higher efficiency in infiltrating and crossing the BBB. It was also reported that such NPs showed promising prospects as brain delivery carriers and brain imaging probes.^182^ More recently, Klok et al.^183^ developed NP-decorated T cells capable of migrating across the BBB independent of any specific antigen and under static conditions and physiological flow. As shown in Figure 7, they were formed by activating effector/memory CD4^+^ helper T cells (CD4^+^ TEM cells) with an approximate diameter of 200 nm and PEG-modified polystyrene NPs using a thiol–maleimide covalent coupling strategy. Besides, the NPs were almost exclusively colocalized with the cell membrane. Noticeably, even the presence of ∼105 NPs per cell did not compromise the ability of the CD4^+^ TEM cells to bind to the intercellular adhesion molecule-1 (ICAM-1) and cross the BBB, despite the loss of NP cargo during diapedesis in several cases.^183^

### 7.2 Poly(lactic-*co*-glycolic acid)-Modified Nanomaterials

Poly(lactic-*co*-glycolic acid) (PLGA) is randomly polymerized and composed of two monomers, lactic acid and glycolic acid. PLGA is biodegradable, biocompatible, and nontoxic, and because of that, PLGA is widely used in pharmaceutical products and other industries. Hence, the exciting properties of PLGA in crossing the BBB were extensively investigated. In 2019, Jeon et al. designed functionalized PLGA NPs, coupled with the vitamin D-binding protein (DBP) through an emulsion diffusion approach, and successfully tested them in AD treatment *in vitro*.^184^ It is known that DBP can greatly reduce amyloid beta (Aβ) peptide aggregation, which is one of the predominant causes of AD. As a result, DBP-PLGA NPs exhibited a better therapeutic effect to impede Aβ-polymerization than the free DBP at the same dose for AD therapy. The DBP-PLGA NPs presented an effective platform for delivering cargo to the CNS due to their impressive abilities in crossing the BBB. In another study, the dendrimer poly(amidoamine) (PAMAM) was conjugated with bovine serum albumin (BSA) by carboxyl activation to develop dendrimer-cationized-albumin (dCatAlb), which was subsequently linked with doxorubicin (DOX)-loaded PLGA NPs to form dCatAlb-pDNP NPs. In this study, the caveolae- and clathrin-mediated process through either endocytosis or absorptive-induced transcytosis was reported to be responsible for the internalization of the NPs. They displayed better BBB penetration via absorptive-induced transcytosis due to the cationic attribute of the BSA and therefore were positively tested to treat glioblastoma successfully.^184^ Recently, Chung et al.^185^ synthesized a biodegradable and biocompatible form of PLGA NPs to be employed for CNS drug delivery. A 29-amino-acid rabies virus glycoprotein (RVG29) was capped on the surface of PLGA, enabling these polymeric NPs to cross the BBB by targeting the nicotinic acetylcholine receptors. And because of such surface modification, the blood residence time of these PLGA NPs was extended, and consequently, the exposure of cargo to the BBB was enhanced too. The RVG29 receptors facilitated the intranasal delivery of RVG29-capped NPs to the brain with quick kinetics.^185^ Cano et al.^186^ developed PEGylated PLGA via a double emulsion approach to “envelop” epigallocatechin-3-gallate (EGCG) and ascorbic acid (AA) for AD therapy. These functionalized NPs had optimum bioavailability and when they were injected intraperitoneally in the mice model of AD, showed significant therapeutic efficacy by enhancing spatial learning and improving memory in the AD mouse model. This presented a safe and effective strategy to increase synaptophysin expression and decrease neuroinflammation in the nervous system.^186^

**Figure 8:**
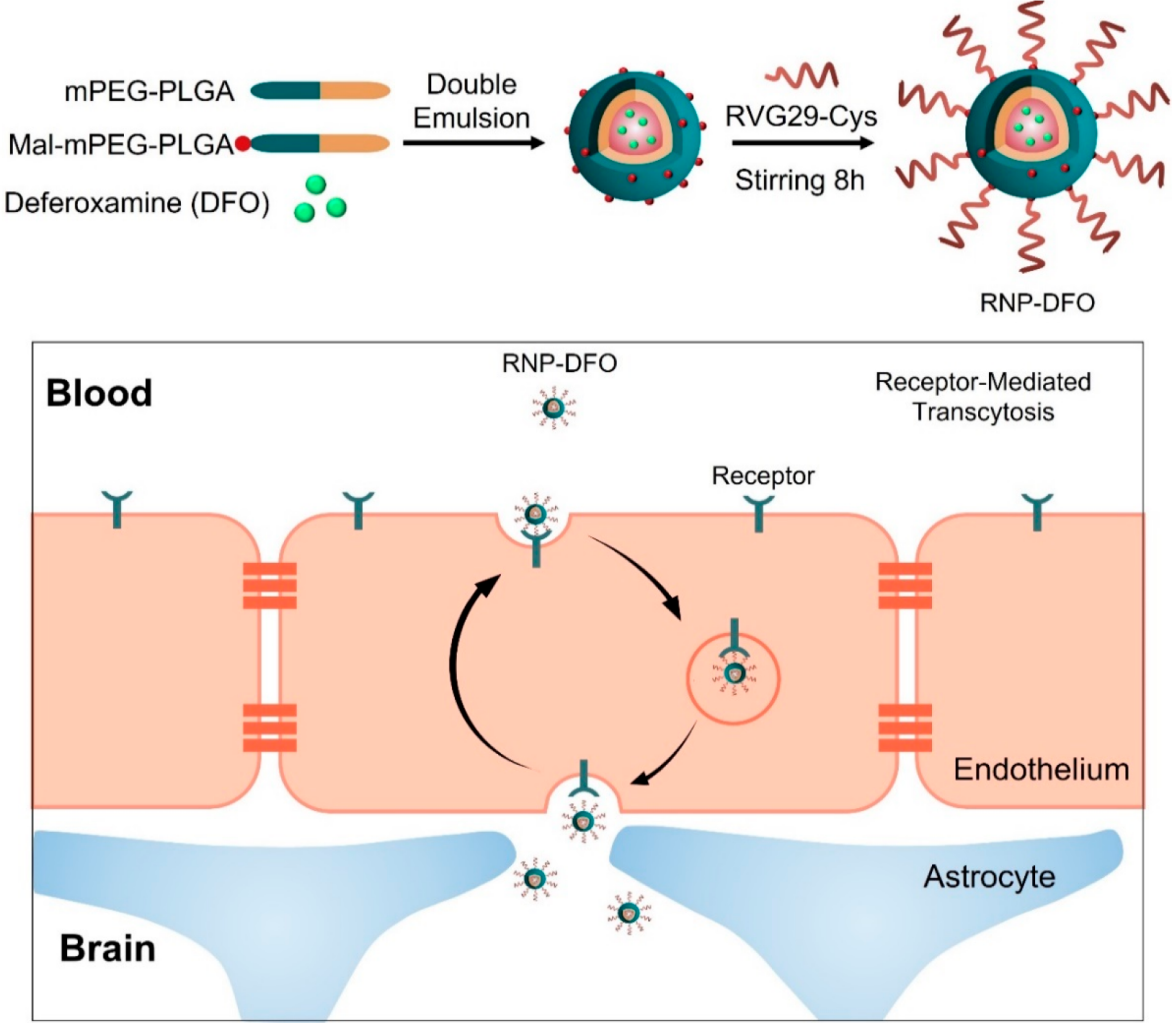
Schematic diagram of the synthesis of RNP-DFO and the proposed mechanism of their penetration into and crossing of the BBB. The brain endothelial cells are bounded by cell surface receptors along with the RVG29 peptide, which facilitates transcytosis of NPs. Reproduced with permission from ref (188). Copyright 2018 American Chemical Society).

Tosi et al.^187^ constructed functionalized PLGA-NPs with angiopep-2 (Ang-2) through the nanoprecipitation approach. Ang-2 is a BBB penetrating peptide, which can bind with the low-density lipoprotein receptor-related protein 1 (LRP1) in the CNS. These NPs displayed the potential tp accumulate in the brain and, hence, presented a promising tool for targeted nanomedicines (Ang-2) in AD. Furthermore, these NPs could be employed as cargo-carriers for delivering various pharmaceutical agents into the CNS parenchyma, therefore significantly expanding the therapeutic possibilities.^187^ In another case, Nie et al. developed an RVG29 peptide-conjugated monomethoxy-PEG (mPEG)-PLGA NP (RNP-DFO) that enabled DFO (an iron chelation drug approved by the FDA for refractory anemia) to circulate for a longer period of time, while also being effectively delivered to the brain.^188^ This demonstrated a specific affinity between the RVG29 peptide and its receptors on the brain’s microvascular endothelium, facilitating the BBB crossing of the NPs into neurons. The delivered cargo (drug) would eliminate surplus cellular iron, triggering the decrease in iron-related oxidative stress resulting from the delivery of the iron chelator DFO across the BBB (Figure 8). Besides, no *in vivo* neuronal apoptosis or other nontargeted adverse effects mediated via NPs were observed. Such a strategy of administering iron chelators for PD treatment could in turn be employed to safely and effectively distribute DFO in the brain.

### 7.3 Lipid-Based Nanocarriers

**Figure 9:**
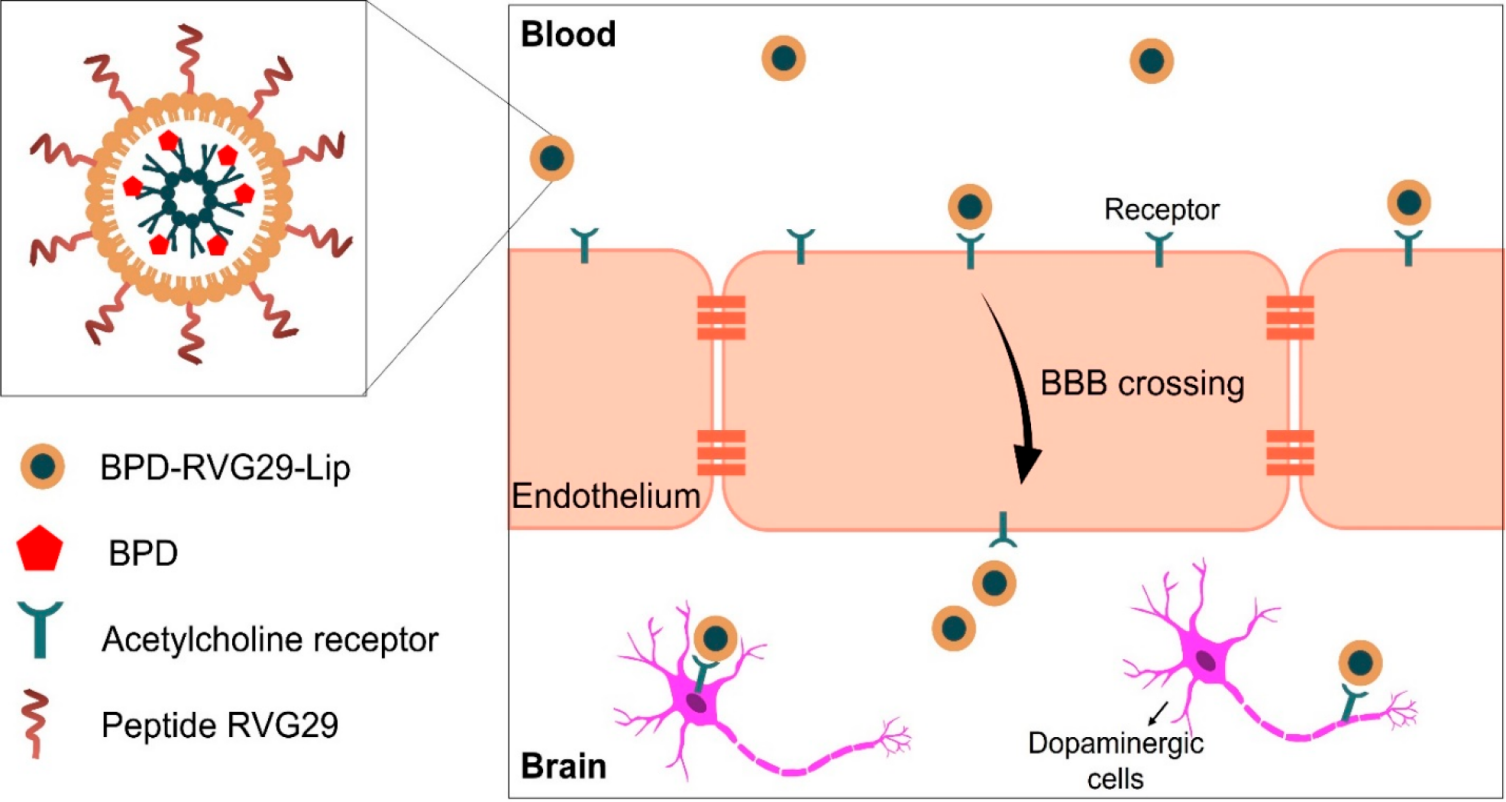
Schematic illustration of RVG29 modified PEGylated liposomes for crossing the BBB and achieving targeted therapy in a PD model. Reproduced with permission from ref (189). Copyright 2018 Elsevier.

Lipids are organic compounds very suitable for acting as a nanocarrier to penetrate the BBB and deliver cargo. Qu et al.^189^ developed therapeutic PEGylated liposomes, which were modified with RVG29 peptide through the maleimide and thiol coupling reaction and loaded with *N*-3,4-bis(pivaloyloxy)-dopamine (BPD). The results confirmed the efficiency of RVG29-modified PEGylated liposomes (RVG29-lip) in crossing the BBB, which was much higher than the PEG-liposomes (Figure 9). This strategy was also implemented to achieve targeted therapy in the PD mouse model.^189^ Rehman et al.^190^ created a brain therapeutic delivery system based on thermoresponsive lipid NPs (TLNs) using the hot melt encapsulation approach. The NPs were packed with paclitaxel (PTX) and employed as a nanocarrier, which was able to infiltrate the BBB and then target glioblastoma cells. These lipid NPs are in the solid state at 37 °C and convert to a liquid state at 39 °C (solid–liquid transition). For this reason, the liquid state could easily be distorted and squeezed past the BBB tight connections. The rapid diffusion of pharmaceuticals from liquid NPs successfully resulted in quicker drug release.^190^ The high permeability and BBB delivery of TLNs were demonstrated via an *in vitro* model achieving the three main elements in CNS drug delivery, BBB crossing, rapid diffusion of the carrier, and fast release of cargo. Jhaveri et al.^191^ developed and reported *in vitro* and *in vivo* investigations using transferrin-targeted liposomes for delivering resveratrol (RSV) to treat GBM. The liposomes were produced via the thin film hydration method, and RSV was loaded into them to synthesize RSV-laden PEGylated liposomes (RSV-L) with the goal of improving the physiochemical characteristics of RSV. The RSV-L NPs with high encapsulation efficiency and a lengthy drug release period were subsequently bonded to transferrin moieties at the end of the liposome chains (Tf-RSV-L) in order to make them “cancer cell specific”. Distinctly, the Tf-RSV-L NPs crossed the BBB by exploiting transferrin as a receptor via receptor-mediated endocytosis.^191^ In addition, Liang et al.^192^ reported a dual-targeting delivery system T7-LDL, which was comprised of low-density lipoprotein particles (LDL) modified with the T7 peptide, a seven-peptide transferrin ligand (TfR). Since the T7 peptide is capable of binding to transferrin receptors on both the BBB and glioma cells and LDL can specifically interact with LDL receptors on brain endothelial cells, the presence of TfR and LDL together could greatly facilitate BBB penetration via receptor-mediated transcytosis. In a follow-up study, the pharmaceutical vincristine sulfate (VCR) was incorporated in the T7-LDL composite by direct hydration of the lipid film and then effectively employed for glioma treatment in both *in vitro* and *in vivo* models.^192^

### 7.4 Polyethylenimine-Modified Nanomaterials

**Figure 10:**
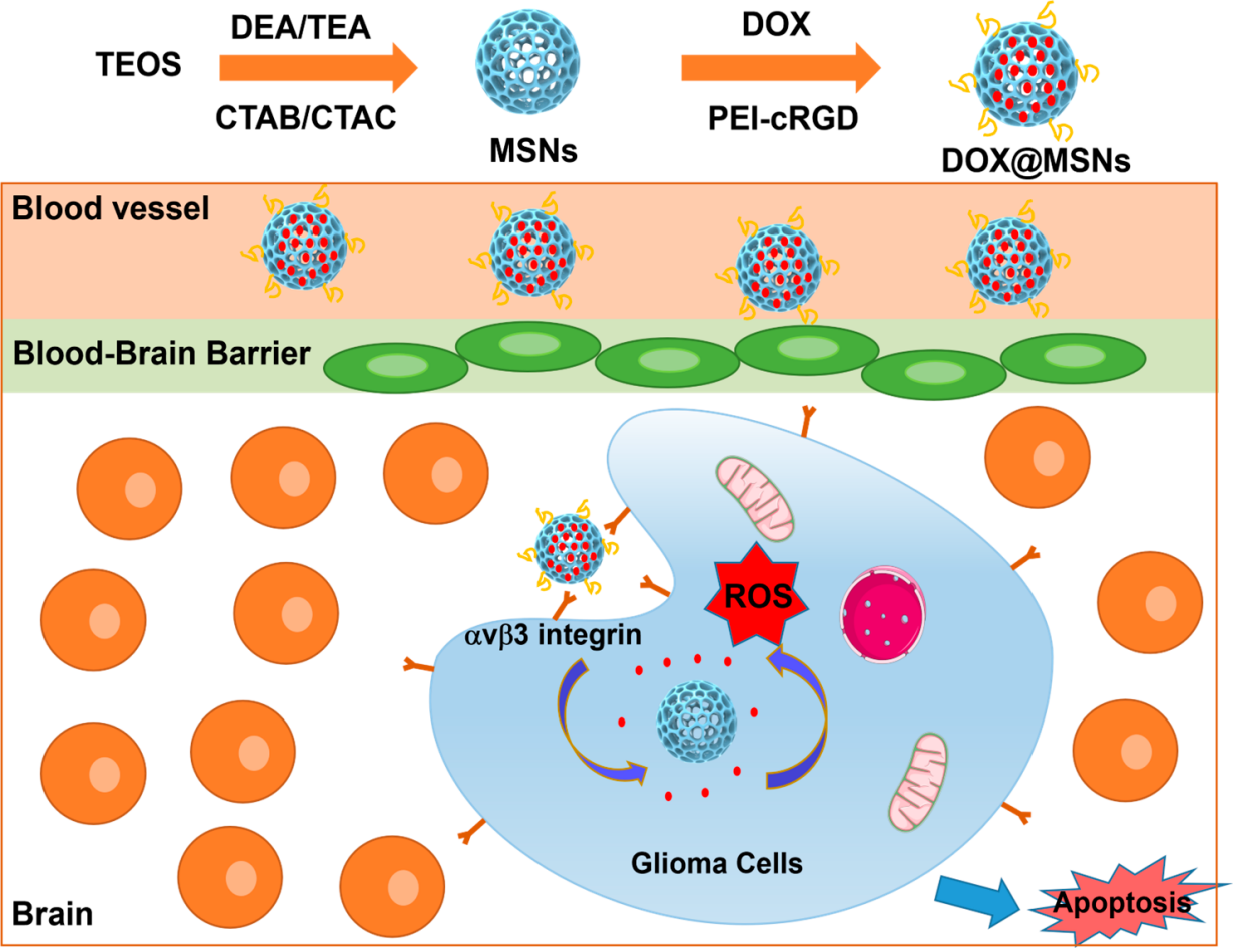
Schematic illustration of MSN NPs enhancing BBB permeability and antiglioma effect. Reproduced with permission from ref (194). Copyright 2016 American Chemical Society.

Polyethylenimine (PEI) is a water-soluble polymer, which could act as a cross-linking agent in various bioapplications. You et al.^193^ developed an RGD (arginine-glycine-aspartate) peptide-modified mesoporous silica-based nanocarrier for delivering benzo[1,2,5]selenadiazole-5-carboxylic acid (BSeC) to the brain for glioma treatment. Because of the presence of RGD in the structure of these nanomaterials, which behaves as an agent to bind to α_v_β_3_ integrin receptors overexpressed on BBB endothelial cells, their BBB permeability was enhanced. The BSeC@MSNs-RGD with PEI coating was found to cross the BBB and disrupt brain cancer U87 VM channels and displayed inhibitory effects on the U87 tumor, therefore validating its anticancer efficacy *in vivo*.^193^ In another example, Chen et al.^194^ developed DOX@MSNs and showed that these NPs were able to penetrate the BBB, target tumor tissues, and contribute to much higher antiglioma efficacy. The sizes of the mesoporous silica nanoparticles (MSNs) were tailored by modulating the hydrolysis rate and polycondensation degree of reactants, with (i) CTAB/CTAC as the template, (ii) TEOS as the silica source, and (iii) DEA/TEA as the base catalyst. The cancer-targeting efficiency of the NPs was enhanced by conjugation with cRGD peptide, which specifically recognizes and binds to U87 cells with a greater expression level of α_ν_β_3_ integrin. Further, antineoplastic DOX (doxorubicin) was loaded into the NPs to inhibit glioma cell growth as well. DOX@MSNs could rapidly enter the cancer cells, resulting in higher drug accumulation in the cytoplasm (Figure 10). DOX@MSNs evoked glioma cell apoptosis through inducing ROS overproduction. DOX@MSNs (∼40 nm) had high permeability across the BBB and disrupted the vasculogenic mimicry of glioma cells by the downregulation of E-cadherin, FAK, and MMP-2 expression. Ideally, this could result in better antiglioma activity with less side effects and toxicities to the surrounding healthy cerebral tissue.^194^ In 2018, Chen et al.^195^ designed HER2@NPs capable of crossing the BBB and efficiently delivering anticancer agents into the brain tissues. The HER2@NPs were constructed using selenium NPs with surface modification of HER2 antibody, which has a higher binding affinity to its receptors overexpressed in glioma cells. HER2@NPs can efficiently deliver both therapeutic (Cu-phen) and diagnostic agents (superparamagnetic iron oxide NPs) across the BBB to the tumor tissues and enhance their impact on brain cancer therapy and magnetic resonance imaging. In this case, Cu-phen is consistently linked to Cu@HER2@NPs when circulating in the blood and could be rapidly released after lysosome escape. From a mechanistic point of view, Cu@HER2@NPs limit the growth, migration, and invasion of U251 glioblastoma via HER2 receptor-mediated endocytosis and trigger DNA damage-mediated p53 signaling pathways. Moreover, Cu@HER2@NPs could cause apoptosis in U251 cells by increasing ROS.^195^

### 7.5 Other Polymeric Nanomaterials

**Figure 11:**
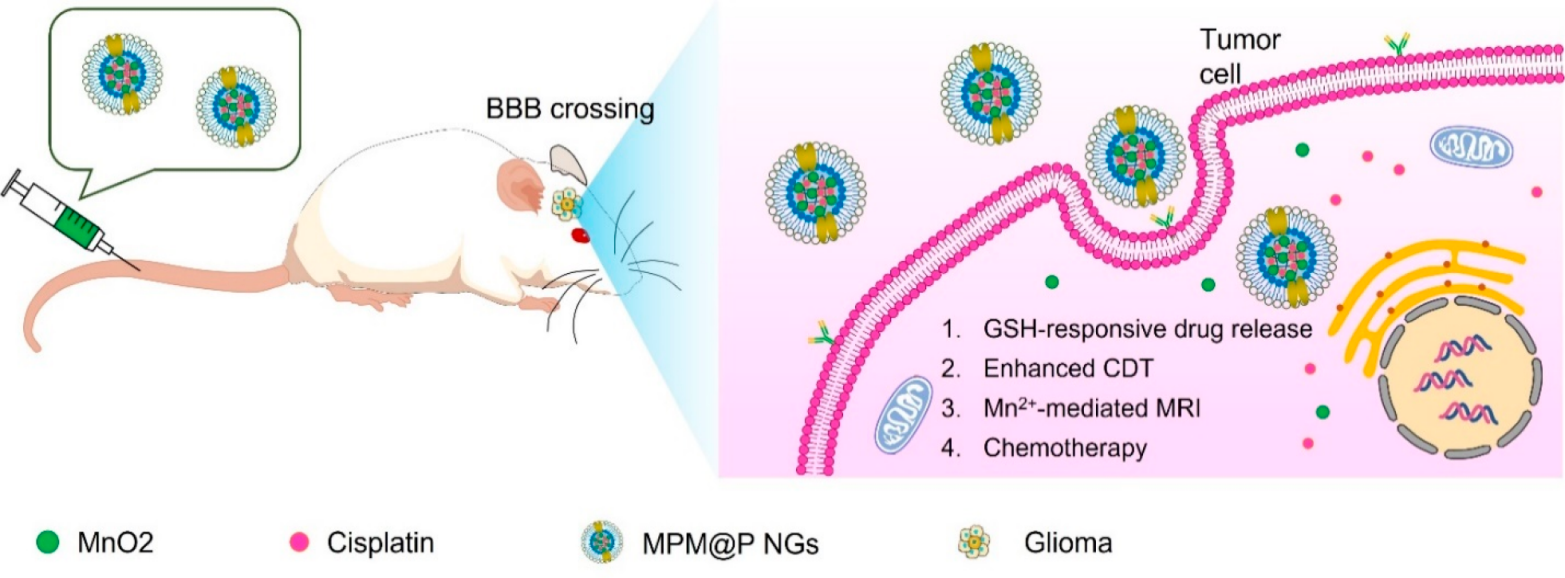
Schematic diagram of the multifunctional polymer nanogels for BBB crossing, MR imaging, and chemotherapy of orthotopic glioma. Reproduced with permission from ref (198). Copyright 2021 American Chemical Society.

Recently, some other drug-related NP constructs were explored and widely utilized for diagnostics and therapeutics. Aguilera et al.^196^ reported magnetic iron oxide NPs, which were coated with carboxymethyl cellulose (CMC) to form Fe_3_O_4_@CMC via electrostatic interaction. These NPs were potential candidates for brain treatment after conjugation with dopamine hydrochloride. They demonstrated that CMC-modified magnetic NPs are able to traverse human lung microvascular endothelial (HLMVE) cells through endocytosis, which was employed as a BBB model.^196^ Furthermore, bioinspired NPs were constructed by Lu et al.^197^ that could effectively cross the BBB and deliver medicine to the CNS. The NPs were formed by a core protein and a thin shell polymerized by 2-methacryloyloxyethyl phosphorylcholine (MPC) monomer and poly(lactide)-*b*-poly(ethylene glycol)-*b*-poly(lactide)-diacrylate triblock copolymer (PLA-based cross-linker). The MPC comprises a choline and acetylcholine analogue, which interacts with nicotinic acetylcholine receptors (nAChRs) and choline transporters (ChTs). The MPC could be actively transported from the bloodstream to the brain via energy-dependent transcytosis. After permeating the BBB, the cross-linker is cleaved, the nanocapsule shells are broken down, and subsequently, the active protein payloads are released into brain tissues.^197^ Shi et al.^198^ utilized the reduction-responsive poly(*N*-vinyl caprolactam) nanogel (PVCL NG) as a carrier. Stable particles (106.3 nm) of colloidal NGs were synthesized by loading MnO_2_ and the chemotherapeutic drug cisplatin. Subsequently, the macrophage membrane was externally coated, and the macrophage membrane-based biomimetic multifunctional responsive drug-carrying nanogel (MPM@P NGs) was developed (Figure 11). This enabled them to efficaciously achieve the combined treatment of *in situ* glioma with real-time MR imaging monitoring. The functionalized nanogels were able to block the function of targeted glioma and cross the BBB, exploiting the interaction between macrophage membrane-specific proteins (such as integrin α_4_, β_1_, etc.) and vascular cell adhesion molecule-1 (VCAM-1) at the tumor site. In addition, the *N*,*N*′-bis(acryloyl) cysteamine (BAC) cross-linked PVCL nanogels containing disulfide bonds showed a desirable reduction response to high concentrations of GSH (10 mM) in tumor cells. GSH can break the disulfide bond and disintegrate the nanogels, thus the drug release at tumor sites can precisely be controlled.^198^

In another study, RSV was used for the treatment of oxidative stress induced by excess reactive oxygen species, which is one of the predominant causes of neurological diseases in the CNS. Cui et al.^199^ loaded MSNs with RSV and utilized them as drug delivery nanocarriers after being capped with poly(lactic acid) (PLA) as a gate keeper. They were also modified with low-density lipoprotein receptors (LDLR) as ligand peptides through covalent bonding with PLA. The PLA enables the manipulation of the pharmaceutical release-times due to its biodegradable property. The LDLR peptides facilitated the transcytosis process of MSNs to permeate the BBB via receptor-mediated transcytosis, as evidenced by an *in vitro* BBB model (established by rat brain microvascular endothelial cells and microglia cells using Transwell chambers).^199^ Additionally, Tang et al.^200^ synthesized NK@AIEdots with superior BBB permeability and excellent ability to inhibit glioma growth. The NK@AIEdots were prepared through wrapping a natural killer cell membrane over an aggregation-induced emission (AIE)-active polymeric nano-endoskeleton (AIEdots) PBPTV, which worked as a photothermal agent under 808 nm laser excitation. The NK@AIEdots exhibited an exceptional NIR-II brightness (quantum yield ≈ 7.9% in water), outstanding stability, excellent biocompatibility, and low cytotoxicity. The surface of NK@AIEdots accurately conserved the complexity of the NK membrane (similar to integrin LFA-1 and VLA-4). They could disrupt tight junctions and reorganize actin cytoskeleton to enhance their abilities to cross the BBB. Besides, NK@AIEdots can specifically accumulate in the brain tumors via DNAM-1/NKG2D receptors and inhibit tumor growth greatly via localized hyperthermia induced by NIR light irradiation. Therefore, these NK-cell-mimetic nanorobots proved to be rather effective BBB-crossing drug delivery agents for the treatment of various neuropathologies.^200^

## 8 Challenges, Directions, and Future Perspectives

Although functionalized nanomaterials have exhibited exceptional neurotheranostic prospects because of their desired properties of crossing the BBB, still their safety and efficacy make their clinical translation from bench to bed side challenging. Previous studies have extensively revealed the cytotoxicity, genotoxicity, and immunotoxicity of diverse nanomaterials and demonstrated that their toxicity is related to their attributes, numbers, coatings, shapes, sizes, surface charges, surface areas, core structures, surface modifications, and exposed cell types. Another challenge in this field is understanding of the BBB structural and physiological properties, especially when they are compromised during a pathological event, like tumor growth or chronic diseases. The tight junctions between endothelial cells in the BBB restrict the passage of molecules, including therapeutic agents, into the brain. Additionally, the presence of efflux transporters further hinders drug delivery. Therefore, developing functionalized nanomaterials that can effectively bypass or overcome these barriers should be one of the practical aims. Another one is the absence of targeted delivery. Achieving targeted delivery of functionalized nanomaterials to specific brain regions remains a challenge. The complexity of the compromised anatomy and physiology of the brain (due to the ongoing pathologies) and its diverse cell types make it difficult to achieve precise targeting.

Based on these, the potential research directions could be focused as follows: (1) Advanced nanomaterial design: Developing functionalized nanomaterials with improved properties, such as enhanced stability, biocompatibility, and BBB permeability, is a promising direction. This includes the use of advanced nanomaterials, such as carbon-based nanomaterials, liposomes, or polymeric nanoparticles. (2) Conjugation strategies: combining functionalized nanomaterials with other approaches, such as drug delivery systems or imaging agents, can improve their efficacy and enable multifunctionality. Coating nanomaterials with targeting ligands or encapsulating therapeutic agents within nanocarriers can enhance BBB crossing and targeted delivery. (3) developing noninvasive methods for delivering functionalized nanomaterials across the BBB is an emerging direction. Techniques such as focused ultrasound, magnetic targeting, or intranasal delivery offer potential noninvasive strategies for enhancing BBB permeability.

Furthermore, it is undeniable that multifunctional nanomaterials as well as various form of nanodelivery platforms will be important paths for the future progress in prevention, diagnostics, therapeutics, and prognostics.^201^ Among numerous strategies, one is a multidisciplinary path to create multifunctional nanosystems that (i) are responsive to the cellular microenvironment, (ii) are capable of safely crossing the BBB and reaching the neuronal parenchyma, (iii) are capable of precisely targeting the cell and tissues of interest, (iv) have optimum capabilities for safe drug delivery, (v) allow effective drug release, and (vi) have acceptable minimum side effects. Certainly, imaging modalities, like MR imaging, positron emission tomography imaging, and optical and photoacoustic imaging could also be employed to develop, boost, and expand such systems. For instance, upconversion nanoparticles (UCNPs) have a wide range of biological applications^168,201−223^ and could be introduced for tracking and coupling polymer luminescent research. Future investigations could take advantage of the unique characteristics of the nanomaterials for combining diagnosis and therapies by creating and integrating various functional groups within one nanosystem to achieve highly sensitive and specific tasks. Imaging-guided targeted therapy is one outstanding example, though it is still in the early stages. Such integrated nanosystems are expected to provide a platform for the efficient management of CNS diseases. It is also expected that biomedical research based on nanomaterials will expand into other mental disorders and psychiatric imaging such as schizophrenia, depression, and anxiety disorders, rather than mainly focusing on brain tumors, neurodegenerative diseases, stroke, and epilepsy. Moreover, innovative functionalized nanomaterials are also demanded to be developed for delivering diverse cargos and therapeutic agents such as antibiotics. This could in turn optimize the delivery, application, dosing, targeting, and consequently, future resistance of the drug.

## Vocabulary

homeostasis: the process of maintaining a constant internal environment within the body
caveolin: a main integral protein for caveolae
hypoxia: low levels of oxygen in the body tissues
enhanced permeability and retention: a property that the appropriate sizes of nanoparticles tend to accumulate in tumor tissue much more than in normal tissues
clathrin: the principal constituent of a polyhedral protein lattice that coats cell membranes
amphiphilicity: the property of some molecules to have an affinity to two phases, polar solvent phase and hydrophobic phase
upconversion nanoparticles: a unique class of optical nanomaterials doped with lanthanide ions featuring a wealth of electronic transitions within the 4f electron shells
protein corona: the presence of a protein layer on the nanoparticle surface.

## Abbreviations

BBB: blood–brain barrier
CNS: central nervous system
NP: nanoparticle
EC: endothelial cell
TJ: tight junction
AMT: adsorptive-mediated transcytosis
RMT: receptor-mediated transcytosis
PLGA: poly(lactic-*co*-glycolic acid)
PEI: polyethylenimine
MSN: mesoporous silica nanoparticle
